# Understanding the Genetic Basis of Spike Fertility to Improve Grain Number, Harvest Index, and Grain Yield in Wheat Under High Temperature Stress Environments

**DOI:** 10.3389/fpls.2019.01481

**Published:** 2019-11-29

**Authors:** Sumit Pradhan, Md Ali Babar, Kelly Robbins, Guihua Bai, Richard Esten Mason, Jahangir Khan, Dipendra Shahi, Muhsin Avci, Jia Guo, Mohammad Maksud Hossain, Madhav Bhatta, Mohamed Mergoum, Senthold Asseng, Paul St. Amand, Salvador Gezan, Byung-Kee Baik, Ann Blount, Amy Bernardo

**Affiliations:** ^1^Department of Agronomy, University of Florida, Gainesville, FL, United States; ^2^School of Integrative Plant Science, Section of Plant Breeding and Genetics, Cornell University, Ithaca, NY, United States; ^3^USDA-ARS, Manhattan, KS, United States; ^4^Crop, Soil, and Environmental Sciences, University of Arkansas, Fayetteville, AR, United States; ^5^Department of Agronomy, University of Wisconsin, Madison, WI, United States; ^6^Department of Crop and Soil Sciences, University of Georgia, Griffin, GA, United States; ^7^Agricultural and Biological Engineering, University of Florida, Gainesville, FL, United States; ^8^School of Forest Resources and Conservation, University of Florida, Gainesville, FL, United States; ^9^USDA-ARS, Wooster, OH, United States; ^10^North Florida Research and Education Center, Quincy, FL, United States; ^11^Department of Plant Pathology, Kansas State University, Manhattan, KS, United States

**Keywords:** single nucleotide polymorphisms, genotyping-by-sequencing, marker-trait associations, quantitative trait loci, genome-wide association study, spike fertility, spike harvest index, marker-assisted breeding

## Abstract

Moderate heat stress accompanied by short episodes of extreme heat during the post-anthesis stage is common in most US wheat growing areas and causes substantial yield losses. Sink strength (grain number) is a key yield limiting factor in modern wheat varieties. Increasing spike fertility (SF) and improving the partitioning of assimilates can optimize sink strength which is essential to improve wheat yield potential under a hot and humid environment. A genome-wide association study (GWAS) allows identification of novel quantitative trait loci (QTLs) associated with SF and other partitioning traits that can assist in marker assisted breeding. In this study, GWAS was performed on a soft wheat association mapping panel (SWAMP) comprised of 236 elite lines using 27,466 single nucleotide polymorphisms (SNPs). The panel was phenotyped in two heat stress locations over 3 years. GWAS identified 109 significant marker-trait associations (MTAs) (p ≤ 9.99 x 10−5) related to eight phenotypic traits including SF (a major component of grain number) and spike harvest index (SHI, a major component of grain weight). MTAs detected on chromosomes 1B, 3A, 3B, and 5A were associated with multiple traits and are potentially important targets for selection. More than half of the significant MTAs (60 out of 109) were found in genes encoding different types of proteins related to metabolism, disease, and abiotic stress including heat stress. These MTAs could be potential targets for further validation study and may be used in marker-assisted breeding for improving wheat grain yield under post-anthesis heat stress conditions. This is the first study to identify novel QTLs associated with SF and SHI which represent the major components of grain number and grain weight, respectively, in wheat.

## Introduction

Wheat (*Triticum aestivum L.*) is one of the major food crops worldwide and is grown on more than 218 million hectares of land with an average grain yield (GY) of 3.3 t ha^−1^ (FAOSTAT, 2016). Environmental constraints, especially high temperature and drought stress are serious threats to wheat production (Pradhan et al., 2012). Lobell et al. (2011) reported a 5.5% decline in world wheat production since 1980, due to increase in global mean temperature. High temperature can decrease GY at any developmental stage but is particularly damaging during anthesis and grain filling period as a result of floret abortion, reduced grain weight and number, and accelerated maturity (Araus et al., 2003; Easterling and Apps, 2005; Farooq et al., 2011). A trend of global warming is expected to continue in the future, increasing temperature up to 2°C by 2050, which may result in further yield losses (IPCC, 2013; Asseng et al., 2015). Therefore, to keep pace with increasing global population, genetic progress of wheat yield will have to increase from 0.3 to 1.1% per year by 2050 under a changing climate including heat stress (Bruinsma, 2011; Asseng et al., 2015).

Genetic improvement of yield is mainly attributed to better partitioning of photosynthetic products i.e., optimizing harvest index (HI)—the GY over the total produced above-ground biomass (Foulkes et al., 2011). The systematic increase in HI, partitioning of assimilates to grain versus non-grain biomass, has a theoretical upper limit of 65% in wheat (Austin et al., 1980; Foulkes et al., 2011). Stable expression of HI at values of 55% and above would deliver a step change (∼20%) in yield potential, given that the current average HI is ca. 0.45–0.51 in spring wheat and 0.50–0.55 in winter wheat (Foulkes et al., 2011). However, limited knowledge on its genetic basis prevents a further increase of HI to its potential (Foulkes et al., 2011). Although, research to enhance wheat photosynthesis has facilitated an increase in biomass, the increased biomass has not fully contributed to yield due to sink limitation (Acreche and Slafer, 2009; Foulkes et al., 2011; Aisawi et al., 2015). Therefore, strategies to improve grain number per unit area is one of the most important avenues in the genetic improvement of HI and yield potential (Foulkes et al., 2011). In wheat, grain number is a product of spike dry weight and grain number per unit of spike chaff weight, which is an indicator of spike fertility (SF). In recent years, multiple studies have reported a strong positive association between SF and grain number m^−2^ in spring wheat (Acreche et al., 2008; Fischer, 2011; Abbate et al., 2013) and winter wheat (Shearman et al., 2005), thus confirming SF as a candidate trait to overcome sink limitations. Even though genetic gain in GY has been attributed to increasing the number of grains per unit area, it is also important to increase grain weight potential while minimizing a possible trade-off between grain per unit area and grain weight (Sadras, 2007; Calderini et al., 2013). One of the potential traits to increase grain weight is spike harvest index (SHI), a major component of grain weight, calculated as the ratio of grain weight to spike dry weight. The genetic basis of SF and SHI is not clearly understood yet. Identifying novel genetic loci associated with these traits has the potential to detect markers that are functionally linked to GY and HI, and its components which could be used to facilitate marker-assisted breeding for improving GY in wheat.

Association mapping is a powerful approach that utilizes genetic diversity and historical recombination events to provide a high resolution of trait-linked loci (Sukumaran and Yu, 2014). Currently, limited information from genome-wide association studies (GWAS) are available for SF and SHI in wheat, particularly under hot environments. Recently, the International Wheat Genome Sequencing Consortium (IWGSC) published a full chromosome-anchored assembly which allows more precise curation of marker trait associations (MTAs) identified by GWAS. In this study, GWAS was performed on 236 advanced soft wheat germplasm using 27,466 SNPs generated by genotyping by sequencing (GBS). The panel was phenotyped under two heat stressed locations over 3 years. The objectives of this study were: i) to identify novel MTAs linked to GY, SF, SHI, and five other yield-related traits under hot environments, and ii) to identify candidate genes for these MTAs and investigate their underlying function.

## Materials and Methods

### Germplasm, Site Description, and Experimental Design

**Figure 1:**
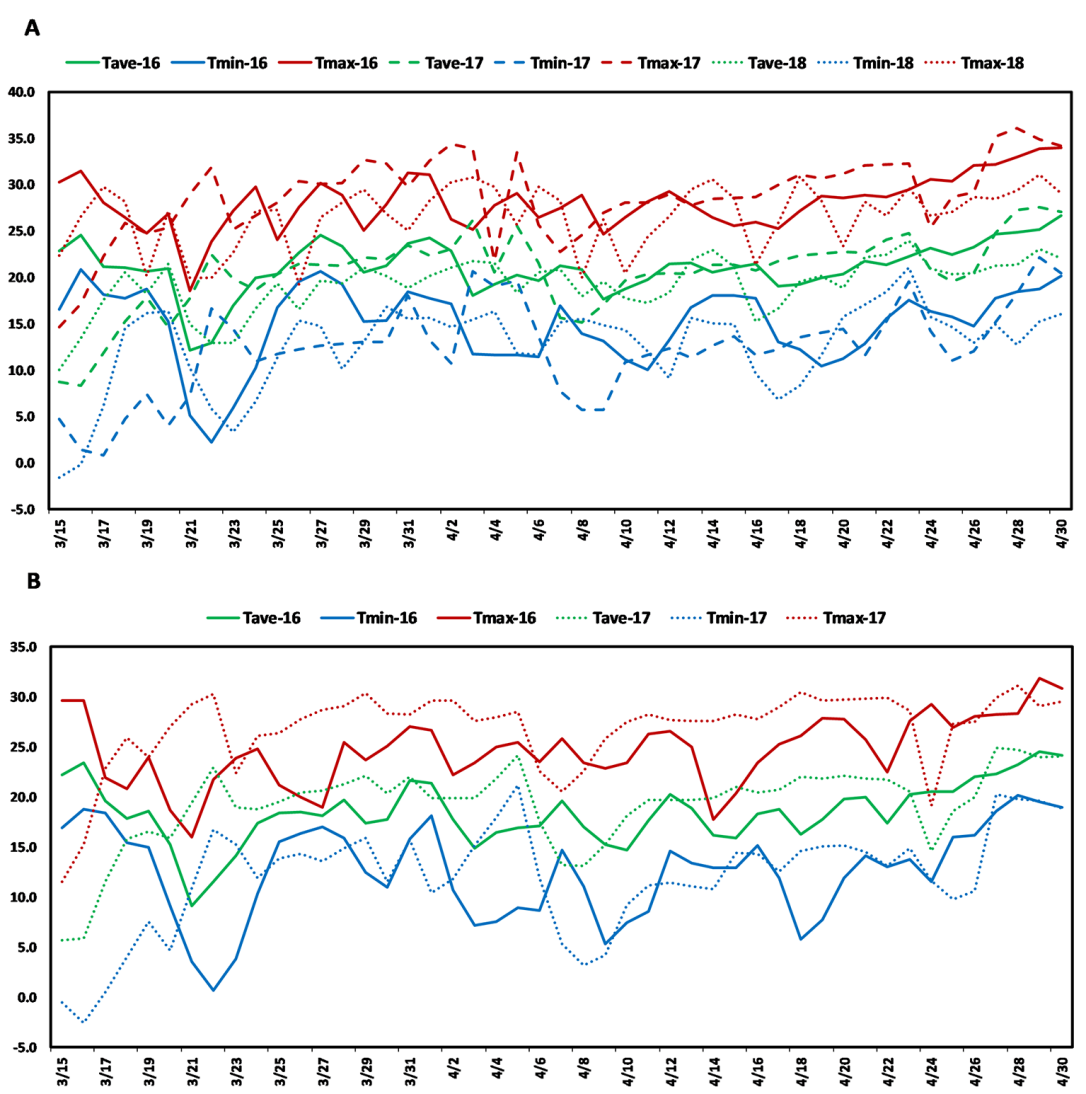
Weather graph showing daily maximum (red), average (green), and minimum (blue) temperatures during grain filling period in **(A)** Citra, FL and **(B)** Quincy, FL. Soft Wheat association mapping panel (SWAMP) was planted three seasons in Citra (2015/2016, 2016/2017, 2017, 2018) and two seasons in Quincy (2015/2016, 2016/2017).

The present study used a soft red facultative wheat association panel consisting of 236 elite lines that are well adapted to the warm and humid south and southeastern regions of the USA. These lines were developed by public and private soft wheat breeding programs in the south and southeastern USA and this collection is hereafter referred to as the Soft Wheat Association Mapping Panel (SWAMP). The SWAMP was evaluated in five yield trials at two heat stress locations in Florida: Citra and Quincy. In Citra, the SWAMP was evaluated in three growing seasons: 2015–2016 (29.407215 ˚N, −82.176 ˚W, elevation = 23 m), 2016–2017 (29.405701 ˚N, −82.175818 ˚W, elevation = 23 m), and 2017–2018 (C18, 29.403853 ˚N, −82.17429 ˚W, elevation = 23 m). In Quincy, the SWAMP was evaluated in two growing seasons: 2015–2016 (30.546202 ˚N, −84.59533 ˚W, elevation = 76 m) and 2016–2017 (30.549658 ˚N, −84.59835 ˚W, elevation = 76 m). Citra had moderate precipitation (212–447 mm), high humidity, and frequent episodes of high temperatures (>30˚C) during the grain filing stages (**Figure 1A**, **Table S3**). Quincy had high precipitation (582–625 mm), high humidity, and comparatively fewer episodes of high temperatures (>30˚C) during the grain filing stages (**Figure 1B**, **Table S3**).

All yield trials were planted in six row plots (3 m length x 1.5 m width) at the rate of 100 kg h^−1^. The SWAMP was planted in a randomized augmented block design (Federer and Raghavarao, 1975) in all trials with 236 un-replicated entries and three repeated check varieties (SS8641, PI 674197; AGS2000, PI 656845; Jamestown, PI 653731). Pesticides were sprayed for management of local diseases, weeds, and insects as required. Fertilizer and irrigation were applied based on plant growth stages and field moisture condition to avoid any water or nutrient limitations, respectively. Planting dates were delayed to late December to increase post-anthesis heat stress conditions.

### Phenotypic Trait Measurement

Eight phenotypic traits were evaluated in this study. GY was calculated by dividing total grain weight from each plot by the plot area, adjusted to 12% moisture and expressed in kg ha^−1^. Days to heading (GS 59), days to anthesis (GS65), and days to physiological maturity (GS90) were determined using the Zadoks scale (Zadoks et al., 1974). Grain filling rate (GFR) was calculated as: GY/(days to physiological maturity–days to anthesis). Thousand grain weight (TGW) was calculated by counting 1,000 kernels in a seed counter (Seedburo Equipment Co., Chicago, IL) and weighing. Grain number (GN) was obtained by dividing total grain weight from each plot by individual grain weight (= TGW/1000). Spike number m^−2^ (SPK) was determined by counting number of spikes in a 0.5 m^2^ area from middle rows and converted to m^2^. HI was determined as the ratio of grain weight m^−2^ to above ground dry matter m^−2^. Ten spikes were sampled randomly from each plot at physiological maturity, dried for 72 h at 60°C, and threshed to determine chaff weight (the non-grain part of a spike), calculated as the difference between total spike dry weight and spike grain weight. SF was measured as a ratio of GN m^−2^ to chaff weight m^−2^ (Abbate et al., 2013). SHI was calculated as the ratio of grain weight of 10 spikes to total spike dry weight.

### Phenotypic Analysis

Combined analysis of variance (ANOVA) was conducted assuming a mixed linear model. The “lme4” package (Bates et al., 2014) and the R software program (v3.5.1, R Development Core Team) were used to estimate the best linear unbiased estimates (BLUEs) assuming a fixed genotypic effect (all other effects wererandom):

$$Y_{\mathrm{ijk}}=\mu +G_{i}+\;E_{j}+\;GE_{\mathrm{ij}}+\;B_{k}{\left(E\right)}_{j}+\;\varepsilon_{\mathrm{ijk}}$$

where the phenotypic response (Yijk) is a function of the overall mean (µ), ith genotype (Gi), jth environment, genotype-environment interaction (G_Eij_), kth block (B_k_) nested within the jth environment (E_j_), and the residual error (ε_ijk_).

Each measured trait was adjusted using days to heading as a covariate. BLUEs for each location were also calculated separately and therefore will be discussed hereafter as BLUEC (BLUE values estimated from Citra), BLUEQ (BLUE values estimated from Quincy), and BLUEA (BLUE values estimated from all environments). Broad sense heritability was calculated assuming random genotypic effect (all other effects were random) and was obtained by:

$$H^{2}=\frac{\sigma_{G}^{2}}{\sigma_{G}^{2}+\sigma_{\frac{\mathrm{GxE}}{n}}^{2}+\sigma_{\frac{E}{n}}^{2}}$$

where H2, broad-sense heritability estimate; σ^2^G, genetic variance; σ^2^G × E, genotype-by-environmental variance; σ^2^E, residual variance; and n, number of environments.

Pearson’s correlations were calculated from BLUEs in R using the “corrplot” package (v3.5.1, R Development Core Team). Pearson's correlation coefficient (r) was used to determine the direction and magnitude of measured traits. Associations between traits were also explored in PC biplot analysis using the package “factoextra” in R (Kassambara and Mundt, 2016).

### Single Nucleotide Polymorphism Genotyping

High quality DNA was isolated from freeze-dried, powdered leaf tissue (∼100 mg) collected from 2-week-old plants using a modified cetyltrimethylammonium bromide (CTAB) protocol (Saghai-Maroof et al., 1984). The GBS libraries were prepared using MspI and PstI-HF restriction enzymes (Poland et al., 2012). The libraries were pooled together in 96-plex and sequenced in an ion torrent proton sequencer (Thermo Fisher Scientific, Waltham, MA, USA) following manufacturer's instructions at the USDA Central Small Grain Genotyping Lab, Kansas State University, Manhattan, KS, USA.

SNP calling was performed using the TASSEL v5.0 GBS v2.0 discovery pipeline (Bradbury et al., 2007). Reads were aligned to the IWGSC reference genome (Appels et al., 2018). Markers were filtered based on the criteria of minor allele frequency (MAF >5%) and missing data (<20%).

### Linkage Disequilibrium and Population Structure Analysis

Coefficients of linkage disequilibrium (r^2^) were plotted against a range of physical distance (bp) for the whole genomes in R (v3.5.1, R Development Core Team) using the “LDcorSV” package (Desrousseaux et al., 2017). LOESS regressions of mean r^2^ between pairs of SNPs were sampled at the ranges of 30,000, 40,000, and 50,000 bp. The intersection between critical value (r^2^ = 0.2) and LOESS line was considered as the distance beyond which linkage disequilibrium (LD) starts to decay. Population structure was observed using discriminant analysis of principal components (DAPC, “adegenet” package, R Development Core Team, 2013) (Jombart et al., 2010). The Bayesian information criterion (BIC) score provided by DAPC was used to infer the best number of genetic groups supported by the results. The principal components analysis was then performed using “prcomp” (of the “stats” package in R) to investigate the genetic differentiation among and within groups.

### Genome-Wide Association Study and Gene Annotation

A genome-wide association study was conducted using the FarmCPU (fixed and random model circulating probability unification) model executed in the Genome Association Prediction Integrated Tool (GAPIT) in R (Lipka et al., 2012). FarmCPU is a recently developed algorithm which uses a random effect model (REM) and a fixed effect model (FEM) iteratively to eliminate false negatives, control false positives, and prevents model overfitting (Liu et al., 2016; Arora et al., 2017). GWAS was performed using three BLUE datasets (BLUEC, BLUEQ, BLUEA) for each trait to identify significant MTAs in the SWAMP. The first three principal components were used as covariates by observing model fit in Q-Q (quantile-quantile) plots, and kinship was determined using FarmCPU (Liu et al., 2016). A uniform value of −log10p = 4.00 (p = 9.99 x 10^−5^) was used as the cut-off to define significant MTAs by looking at Q-Q plots to identify the deviation of the observed test statistics values from the expected test statistics values (Sukumaran et al., 2012; Sukumaran et al., 2015). In addition, a threshold value of −log10p = 3.50 was used as the cut-off to detect pleiotropic markers associated with SF. Manhattan plots were constructed in R using the “qqman” package (Turner, 2014). Candidate genes associated with significant MTAs and their annotation were identified using the IWGSC reference genome (RefSeq v1.0) (Appels et al., 2018). Potential genes were further investigated using past literature for their association with phenotypic traits under heat stress.

## Results

### Phenotypic Analyses

There was significant genotypic variation in the SWAMP for all measured traits in Citra, Quincy, and the combined analysis (**Table S1**) representing diverse genetic backgrounds of the SWAMP. Environments (growing years and location) and their interactions were all significant (P<0.05) determinants of phenotypic traits (**Table S1**). The days to heading ranged from 98 to 119 days with mean of 108 days and days to maturity ranged from 130 to 150 days with mean of 141 days in Citra, and 129–150 days with mean of 139 days in Quincy (data not shown). The GY ranged from 1,469–6,460 kg h^−1^ with mean yield of 3,518 kg h^−1^ in Citra, and 1,780–8,639 kg h^−1^ with mean yield of 5,025 kg h^−1^ in Quincy (**Table 1**). Moderate to high heritability was estimated for the traits with the highest heritability of 0.83 for TGW (**Table 1**).

**Table 1:** Summary of adjusted means of eight phenotypic traits for the soft wheat association mapping panel (SWAMP) using data obtained from Citra, Quincy, and combined analysis.

Traits	Citra		Quincy		Combined		*H*^2^
	Mean	Range	Mean	Range	Mean	Range	
**SF**	93.9	38.7–157.2	104.1	30.9–201.1	97.9	58.6–150.3	0.51
**GY**	3,518.2	1,468.9–6,459.3	5,025	1,779.7–8,638.9	4,128.8	2,308.8–6,239.4	0.56
**GN**	11,480.4	4,652–20,969.3	15,470.7	6,914.2–27,563.8	13,083.9	6,636.2–20,089.7	0.42
**TGW**	31.1	20.3–44.1	34.1	17.9–56.9	32.3	21.2–47.2	0.83
**GFR**	118.5	53–208.6	161.3	64.6–353.1	136	73.7–219.9	0.45
**SPK**	327.2	205.3–506	380.6	188–532.6	348.3	212.9–466.2	0.46
**SHI**	74.7	51–87.2	75.7	54.3–86.4	75.1	59.1–83.7	0.38
**HI**	32.8	11.7–54.6	33.5	13.7–52.8	33.1	15.6–48.2	0.67

SF, spike fertility (grains g^−1^ chaff weight); GY, grain yield (kg h­); GN, grain number m^−2^; TGW, thousand grain weight (g); GFR, grain filling rate (kg h^−1^ days); SPK, number of spikes m^−2^; SHI, spike harvest index; HI, harvest index; H², broad-sense heritability for adjusted means across all environments.

Principal components (PC) analysis showed that PC1 and PC2 explained 72.9, 66, and 71.5% of the total variation for phenotypic trait data from Citra, Quincy, and the combined analysis (**Figure S1**). In the PC biplot, two distinct groups were observed: cluster 1 (SF, GY, GN, HI, SPK, and GFR) and cluster 2 (TGW and SHI). SF was associated with GY, GN, HI, and GFR whereas SHI was associated with TGW and the result was consistent in BLUEA, BLUEC, and BLUEQ (**Figure S1**). PC analysis groupings of phenotypic traits was further supported by Pearson correlation coefficients (**Figure S2**). In all three environments, SF was positively correlated with GY (ranging from 0.37 to 0.46), GN (0.59 to 0.67), GFR (0.37 to 0.43), and HI (0.24 to 0.45). SHI showed consistent positive correlations with TGW (0.44 to 0.55). Moreover, SHI was also positively correlated with SF (0.32 to 0.41) and GY (0.24 to 0.49) (**Figure S2**). Days to heading and days to maturity showed significant negative correlation with GY (−0.49 and −0.45), TGW (−0.47 and −0.46), GFR (−0.36 and −0.39), SHI (−0.26 and −0.21), and HI (−0.66 and −0.64), (data not presented in table). However, SF demonstrated non-significant correlations with days to heading (−0.08) and days to maturity (−0.05).

### Genetic Data, Linkage Disequilibrium Decay, and Population Structure

**Figure 2:**
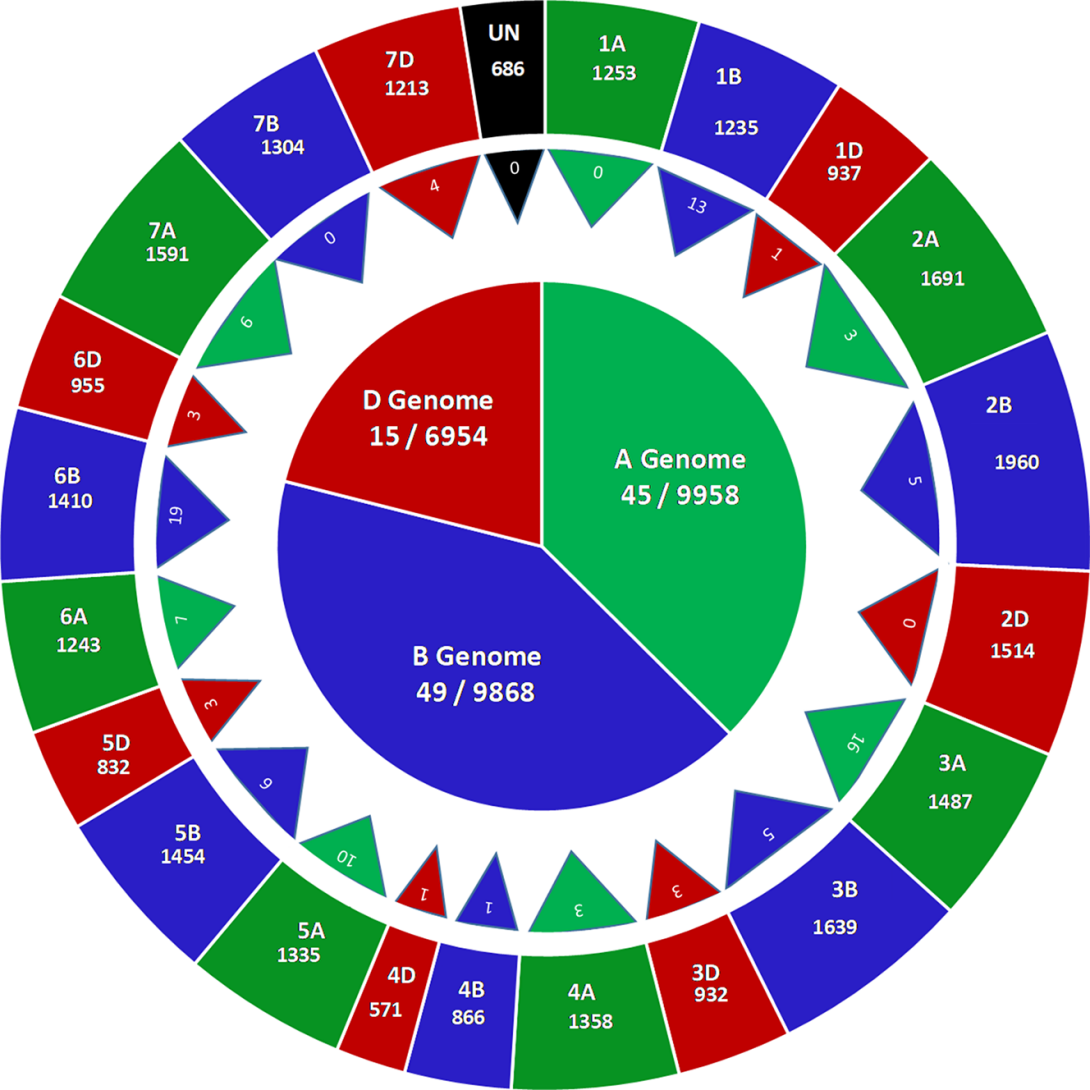
Genome wide distribution of 27,466 single nucleotide polymorphisms (SNPs) markers (outer circle), number of marker trait associations across 21 chromosomes (triangles) and number of marker trait associations across three genomes (inner circle) in a soft wheat association mapping panel (SWAMP).

GBS of 236 accessions in the SWAMP produced 27,466 SNP markers that were used for GWAS. These SNP markers were distributed throughout the A (9958, ∼36%), B (9,968, ∼36%), and D (6,954, ∼25%) genomes (**Figure 2**). A total of 686 SNPs where found on unplaced scaffolds and thus were classified as unmapped SNPs. Chromosome 2B had the highest number of SNPs (1,960) and chromosome 4D had the lowest (571) (**Figure 2**). In order to determine the approximate marker density required for GWAS, LD was computed using the “LDcorSV” package in R. The LD decay below the line of critical value (r^2^ = 0.2) was estimated at 1,182, 1,920, and 2,916 bp for ranges of 30,000, 40,000, and 50,000 bp, respectively, across the whole genome (**Figure S4**). The magnitude of change in LD decay between a sample range of 30,000–50,000 bp was 1,734 bp. Population structure was investigated to avoid false positive associations in GWAS (Sukumaran and Yu, 2014). PC analysis revealed substantial admixture among lines in the SWAMP, with the first and second PC explaining only 4.7 and 3.1% of the total genotypic variance, respectively (**Figure S5B**). The simulation result using DAPC suggested three genetic groups (K = 3) (**Figure S5A**). The first, second and third group contained 49, 144, and 43 lines, respectively, and were roughly divided according to geographical locations.

### Marker-Trait Association Analysis

**Figure 3:**
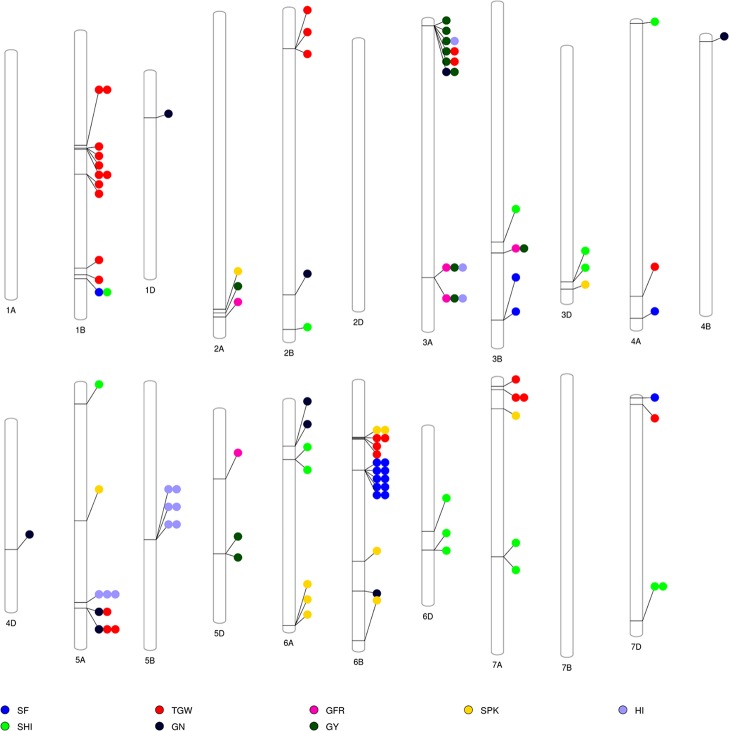
Overview of significant markers trait associations identified on each chromosome for eight phenotypic traits obtained from GWAS using BLUEC, BLUEQ, and BLUEA datasets in a soft wheat association mapping panel (SWAMP). SF, spike fertility (grains g^−1^ chaff weight); GY, grain yield (kg h^−1^); GN, grain number m^−^²; TGW, thousand grain weight (g); GFR, grain filling rate (kg h^−1^ days); SPK, number of spikes m^−^²; SHI, spike harvest index; HI, harvest index.

The GWAS identified novel MTAs for all measured traits and explained high phenotypic variances. The FarmCPU model with kinship and PC scores was used to identify MTAs for each trait using 27,466 GBS-derived SNPs. The SNP markers were uniformly distributed throughout the chromosome of each genome (**Figures 2** and **3**). GWAS was conducted on three datasets: BLUEC (Citra), BLUE Q (Quincy), and BLUEA (combined). We identified 109 significant MTAs for 8 phenotypic traits distributed across 18 chromosomes with phenotypic variations explained (PVE) ranging from 7 to 31% (**Table S2**, **Figures 2** and **3**). We found significant MTAs across all the chromosomes except on chromosome 1A, 2D, and 7B (**Figures 2** and **3**). The highest number of MTAs was detected in BLUEA (39) followed by BLUEC (38) and BLUEQ (32) (**Table 2**, **Figures 2** and **3**). Larger number of MTAs were identified in the A (45 MTAs) and B (49 MTAs) genomes rather than in the D genome (15 MTAs).

**Table 2:** Summary of significant marker–trait associations for eight phenotypic traits in the soft wheat association mapping panel (SWAMP) using data obtained from Citra, FL, Quincy, FL and combined analysis.

Traits	BLUEC	BLUEQ	BLUEA	Total	Chromosomes
**SF**	6	1	8	15	1B, 3B, 4A, 6B, 7D
**GY**	9	2	1	12	2A, 3A, 3B, 5D
**GN**	4	5	1	10	1D, 2B, 3A, 4B, 4D, 5A, 6A, 6B
**TGW**	4	10	14	28	1B, 2B, 3A, 4A, 5A, 6B, 7A, 7D
**GFR**	3	0	2	5	2A, 3A, 3B, 5D
**SPK**	2	7	2	11	2A, 3D, 5A, 6A, 6B, 7A
**SHI**	6	3	7	16	1B, 2B, 3B, 3D, 4A, 5A, 6A, 6D, 7A, 7D
**HI**	4	4	4	12	3A, 5A, 5B
**Total**	38	32	39	109	1B, 1D, 2A, 2B, 3A, 3B, 3D, 4A, 4B, 4D, 5A, 5B, 5D, 6A, 6B, 6D, 7A,7D

SF, spike fertility (grains g^−1^ chaff weight); GY, grain yield (kg h^−1^); GN, grain number m^−2^; TGW, thousand grain weight (g); GFR, grain filling rate (kg h^−1^ days); SPK, number of spikes m^−2^; SHI, spike harvest index; HI, harvest index.

We identified 15 MTAs for SF across three datasets on chromosomes 1B, 3B, 4A, 6B, and 7D for which PVEs ranged from 8 to 11% (**Table 2**, **Table S2**). For SHI, 16 MTAs were identified on chromosomes 1B, 2B, 3B, 3D, 4A, 5A, 6A, 6D, 7A, 7D with PVEs ranging from 7 to 14%. In this study, we detected 12 MTAs for GY on six chromosomes 2A, 3A, 3B, and 5D with PVEs ranging from 14 to 30%. For GN, 10 significant MTAs were distributed on chromosomes 1D, 2B, 3A, 4B, 4D, 5A, 6A, and 6B with PVEs ranging from 12 to 16%. The MTAs identified for TGW (28 MTAs) on chromosomes 1B, 2B, 3A, 4A, 5A, 6B, 7A, and 7D had PVEs ranging from 24 to 31%. For HI, 12 MTAs were detected on chromosomes 3A, 5A, and 5B with PVEs ranging from 22 to 25%. GFR had the fewest MTAs, with only 5 MTAs on chromosomes 2A, 3A, 3B, and 5D with PVEs ranging from 19 to 25%. For SPK, 11 MTAs were located on chromosomes 2A, 3D, 5A, 6A, 6B, 7A with PVEs ranging from 8 to 14% (**Table 2**, **Table S2**).

We found seven pleiotropic SNP markers on chromosomes 1B, 3A, 3B, and 5A across different datasets (**Table 3**). Five out of seven multi-trait markers were associated with GY and either HI, TGW, GN, or GFR. SNP S3A_625236830 was associated with GY, HI, and GFR and had a positive allelic effect on all three traits. SNP S3A_12915079 had a positive allelic effect for GY (151.8) and GN (324.68) and PVEs of 28 and 15%, respectively. SNP S1B_597446331 was associated with SF and SHI and had a negative allelic effect on both traits (**Figure S6A**). Moreover, at significance threshold of -log10(P) ≤ 3.5 we found additional pleiotropic markers associated with SF and other traits (**Table S4**). S1D_108803053 and S2B_692461029 were associated with SF and GN and had a negative allelic effect on both traits (**Figure S6B**; **Figure S6C**). SNP S3A_263436889 was associated with SF, GN, and GY and had a positive allelic effect on all the traits (**Figure S6D**). S5A_605144046 was associated with SF, GN, and TGW and had a positive allelic effect on SF and GN and negative allelic effect on TGW (**Figure S6E**). SNP S7D_1548220 was associated with SF and HI and had a negative allelic effect on both traits (**Figure S6F**).

**Table 3:** List of significant markers associated with multiple phenotypic traits (pleiotropy), in the SWAMP obtained from GWAS using three datasets: BLUEC (Citra), BLUEQ (Quincy), and BLUEA (combined).

SNP	Trait	Dataset	−log10(p)	Effect	PVE
S1B_597446331	SF	BLUEA	4.18	−4.85	0.09
	SHI	BLUEA	4.88	−0.87	0.11
S3A_12525847	GY	BLUEC	4.75	149.89	0.28
	HI	BLUEC	4.29	1.37	0.21
S3A_12554700	GY	BLUEC	5.10	−148.37	0.29
	TGW	BLUEQ	4.55	−0.92	0.28
S3A_12915079	GN	BLUEC	4.60	324.68	0.15
	GY	BLUEC	4.87	151.80	0.28
S3A_625236830	GFR	BLUEC	4.86	8.36	0.25
	GY	BLUEC	4.69	309.94	0.29
	HI	BLUEC	4.63	2.97	0.23
S3B_605293365	GFR	BLUEA	4.38	3.39	0.19
	GY	BLUEA	4.14	123.54	0.26
S5A_605144047	GN	BLUEQ	4.14	643.69	0.12
	TGW	BLUEA	4.10	−1.71	0.30
	TGW	BLUEQ	5.05	−1.64	0.28

SF, spike fertility (grains g^−1^ chaff weight); GY, grain yield (kg h^−1^); GN, grain number m^−2^; TGW, thousand grain weight (g); GFR, grain filling rate (kg h^−1^ days); SHI, spike harvest index; HI, harvest index; PVE, phenotypic variance explained.

### Gene Annotation

Functional annotation of all significant MTAs was carried out using the IWGSC v1.0 sequence assembly. Sixty out of 109 MTAs were anchored within genes with a wide range of functional annotations (**Table S2**). Candidate genes associated with SNPs were investigated for their functions with reference to past literature. We discovered 33 potential candidate genes encoding different classes of proteins that have suggestive roles in response to biotic, abiotic, and metabolic traits, including F-box family proteins, RNA-binding proteins, carboxypeptidases, receptor-like kinases, heat shock proteins, and senescence related proteins (**Table 4**).

**Table 4:** List of potential candidate genes and anchoring markers associated with eight phenotypic traits under hot environment.

Trait	Dataset	SNP	−log10(p)	Effect	PVE	Allele	Gene-ID	Annotation
SF	BLUEA	S1B_597446331	4.18	−4.85	0.09	A/**T**	TraesCS1B01G366100	DUF616 family protein
SF	BLUEA	S3B_768483625	4.24	3.22	0.08	T/**C**	TraesCS3B01G526100	Endo-1,3-beta-glucanase
SF	BLUEC, BLUEA	S6B_238165774	5.15	4.12	0.10	A/**G**	TraesCS6B01G199000	Alpha/beta-Hydrolases superfamily protein
SF	BLUEC	S7D_1548220	4.27	−3.38	0.08	A/**C**	TraesCS7D01G002400	Protein kinase, putative
GY	BLUEC	S2A_726414492	4.16	250.01	0.28	A/**C**	TraesCS2A01G494600	XH/XS domain protein
GY	BLUEC	S3A_12487841	4.06	130.33	0.27	A/**T**	TraesCS3A01G022000	Sulfotransferase
GN	BLUEQ	S2B_692461029	4.64	−618.9	0.13	G/**T**	TraesCS2B01G495700	RNA-binding family protein
GN	BLUEQ	S4B_13299920	4.72	411.91	0.12	T/**C**	TraesCS4B01G018400	Senescence-associated family protein, putative (DUF581)
GN	BLUEQ	S5A_605144047	4.14	643.69	0.12	T/**C**	TraesCS5A01G416300	Inosine-5'-monophosphate dehydrogenase
GN	BLUEQ	S6B_564061696	4.04	401.04	0.12	T/**C**	TraesCS6B01G316000	Cellulose synthase, putative
TGW	BLUEQ, BLUEA	S1B_272880854	4.81	1.46	0.30	C/**G**	TraesCS1B01G159900	RING finger and CHY zinc finger protein
TGW	BLUEA	S1B_280209703	4.36	1.42	0.29	G/**T**	TraesCS1B01G162600	Adenine nucleotide alpha hydrolases-like superfamily protein, putative
TGW	BLUEQ	S1B_343349481	4.11	1.59	0.29	G/**T**	TraesCS1B01G191900	Protein XRI1
TGW	BLUEA	S1B_587501379	4.32	−1.59	0.30	G/**A**	TraesCS1B01G357600	Histone H2B
TGW	BLUEC	S2B_93702429	4.8	−2.52	0.26	C/**A**	TraesCS2B01G125400	Heat shock 70 kDa protein
TGW	BLUEA	S4A_668108005	4	1.40	0.29	A/**G**	TraesCS4A01G391300	wall-associated receptor Kinase-like protein
TGW	BLUEQ, BLUEA	S5A_605144047	5.05	−1.64	0.28	T/**C**	TraesCS5A01G416300	Inosine-5'-monophosphate dehydrogenase
TGW	BLUEA	S6B_152196843	4.05	−1.11	0.30	C/**A**	TraesCS6B01G151200	CASP-like protein
TGW	BLUEQ, BLUEA	S7A_27206610	4.55	1.60	0.31	A/**G**	TraesCS7A01G056500	Transmembrane protein, putative
TGW	BLUEQ	S7D_18808932	4.04	1.15	0.27	C/**T**	TraesCS7D01G036900	Flavonoid 3'-hydroxylase
SPK	BLUEA	S2A_717557255	4.06	−6.61	0.10	G/**A**	TraesCS2A01G479200	F-box family protein
SPK	BLUEQ, BLUEA	S6B_148823518	4.96	−6.53	0.11	A/**G**	TraesCS6B01G147700	ATP-dependent DNA helicase MER3-like protein
SPK	BLUEQ	S6B_484538781	4.29	−5.11	0.08	A/**G**	TraesCS6B01G269100	Lysophospholipid acyltransferase
SPK	BLUEQ	S6B_699027892	4.36	−7.07	0.08	G/**A**	TraesCS6B01G431300	Receptor-like kinase
SHI	BLUEA	S1B_597446331	4.88	−0.87	0.11	A/**T**	TraesCS1B01G366100	DUF616 family protein
SHI	BLUEA	S2B_776888645	4.57	0.80	0.10	C/**A**	TraesCS2B01G591500	Auxin-responsive protein
SHI	BLUEA	S3B_578403246	4.86	−0.92	0.11	C/**T**	TraesCS3B01G365900	Calmodulin-related protein
SHI	BLUEC	S3D_567747621	4.78	−0.71	0.12	G/**T**	TraesCS3D01G463600	Wall-associated receptor kinase 2
SHI	BLUEA	S4A_4156017	4.43	0.92	0.11	G/**A**	TraesCS4A01G006400	Flavin-containing monooxygenase
SHI	BLUEC	S7A_479346340	4.14	−1.20	0.13	A/**C**	TraesCS7A01G329300	High mobility group family
HI	BLUEQ, BLUEA	S5B_421762510	4.01	2.19	0.24	G/**A**	TraesCS5B01G242300	Carboxypeptidase

SF, spike fertility (grains g^−1^ chaff weight); GY, grain yield (kg h^−1^); GN, grain number m^−2^; TGW, thousand grain weight (g); GFR, grain filling rate (kg h^−1^ days); SPK, number of spikes m^−2^; SHI, spike harvest index; HI, harvest index; PVE, proportion phenotypic variance explained.

## Discussion

Post-anthesis heat stress is a common yield-limiting factor for soft wheat in the southeast US and other wheat growing areas (Russell and Van Sanford, 2018). The optimal temperature for wheat during grain filling stages is 15–18°C (Wardlaw and Wrigley, 1994; Porter and Gawith, 1999) and even a small increase above this temperature can have an adverse effect on GY. The daily high temperatures of 25–36°C (**Figure 1**, **Table S3**) was common in Citra, FL and Quincy, FL during anthesis and grain filling period. Even 2 or 3 days of heat (>35°C) during this period can cause severe yield losses (Prasad et al., 2008). These two locations, particularly Citra, had higher temperatures than optimal such that crops were exposed to episodes of heat stress during the grain filling period (**Figure 1**, **Table S3**). Therefore, the MTAs identified in this study can provide important information for improving performance of soft wheat under heat stress environments.

In this study, SF showed significant genotypic variation with moderate broad-sense heritability (0.51) (**Table 1**). Previous studies have also reported similar results for SF not only in advanced breeding lines but also in early generation breeding populations (Abbate et al., 2013; Martino et al., 2015; Mirabella et al., 2016). Foulkes et al. (2011) reported that reduction in chaff to grain ratio (indicator of SF) can potentially contribute (in conjunction with other traits, such as spike partitioning index, internode partitioning index, etc.) to achieving the theoretical HI limit of 65% (Foulkes et al., 2011), which would be an important step toward breaking the yield barrier for modern wheat varieties. SF also showed a strong positive correlation in all environments with GY, GN, GFR, and HI. This was further supported by PC biplot analysis which showed that SF was clustered with GY, GN, HI, and GFR across all environments. The findings of this study aligns with previous studies (Abbate et al., 2013) and indicates that SF can be a promising breeding target for genetic improvement of sink strength, which ultimately can improve HI and yield potential in wheat under different environments (Fischer, 2011; González et al., 2011; Slafer et al., 2015). We identified 15 MTAs for SF in 10 genomic regions on chromosomes 1B, 3B, 4A, 6B, and 7D with PVEs ranging from 8 to 11% (**Table 2**, **Figure S3.1**). This is the first study to report MTAs responsible for SF in soft wheat grown at high temperature and the MTAs identified for SF are potentially novel. Fourteen out of 15 MTAs were located in four genomic locations and their functional annotation suggests their involvement in abiotic stress including heat stress (**Table 3** and **Table S2**). Two MTAs (S3B_768483613 and S3B_768483625) within 15 bp range were found in the gene, TraesCS3B01G526100, which is annotated as 1,3-beta-glucanase. Havrlentová et al. (2016) suggested this gene as having a possible protective mechanism role in oat during heat stress conditions. Five markers on chromosome 6B (S6B_238165759, S6B_238165760, S6B_238165774, S6B_238165775, S6B_238165776) all within 17 bp range were detected in multiple environments suggesting the genetic stability of these MTAs. These MTAs were present within gene TraesCS6B01G199000, which is annotated as an alpha/beta-hydrolase superfamily protein and have been reported as housekeeping genes that have a roles in the breakdown and detoxification of xenobiotics, and also abiotic stress tolerance, cellular metabolite recycling, and processing of external nutrients (Liu et al., 2014; Mindrebo et al., 2016). Another MTA (S7D_1548220) within gene TraesCS7D01G002400 (protein kinase) is also reported to have functional roles in signal transduction pathways during abiotic and biotic stress (Ho, 2015). This MTA had pleiotropic effect for SF and HI (−log10(P) ≤ 3.5) indicating a genetic association between these traits (**Table S4**). Our study identified novel MTAs associated with SF in US soft wheat under hot and humid conditions which contributes to a better understanding of the genetic basis of SF traits in wheat. Upon further validation, these MTAs can be used in future marker assisted breeding programs to overcome sink limitations, improve HI, and ultimately increase yield potential.

We found significant genotypic variation for SHI across all environments with moderate broad sense heritability (0.38) (**Table 1**). PC biplot analysis and correlation coefficients between eight phenotypic traits showed that SHI was positively associated with TGW in all environments, indicating that SHI can be used as an effective component trait to attain high grain weight in wheat. Moreover, SHI was also positively correlated with SF, GY, and HI (**Figure S2**) indicating that an increase of SHI may increase GN and grain weight simultaneously to optimize yield potential. The 16 MTAs identified for SHI on chromosomes 1B, 2B, 3B, 3D, 4A, 5A, 6A, 6D, 7A, and 7D have PVEs ranging from 7 to 14% (**Table 2**, **Figure S3.7**). This is the first study to report MTAs responsible for SHI on soft wheat and these are potentially novel. Six MTAs for SHI detected on chromosomes 1B, 2B, 3B, 3D, 4A, and 7A have gene annotations suggesting their roles in biotic and abiotic stress (**Table 4**). We detected a pleiotropic MTA on chromosome 1B (S1B_597446331) for SHI and SF indicating a genetic association between these traits. This MTA was within gene TraesCS1B01G366100 (DUF616 family protein) whose function is yet to be determined (Liu et al., 2014; Mindrebo et al., 2016). An MTA on chromosome 2B (S2B_776888645) is within gene TraesCS2B01G591500 which is annotated as an auxin-responsive protein. These transcription factors were previously reported in a study carried out on heat tolerance in sorghum (Chen et al., 2017) and thus may have an important role in response to high temperature stress. Two MTAs on chromosome 7A (S7A_479346340 and S7A_479346380) within 40 bp of each other, are present within the gene TraesCS7A01G329300. This gene is annotated as a high mobility group family protein that play important roles in drought and salinity stress (Kwak et al., 2007). Another MTA (S3D_567747620) within gene TraesCS3D01G463600 (wall-associated receptor kinase) was reported in Arabidopsis to have important roles in heavy-metal stress tolerance, pathogen resistance, and cell expansion (Zhang et al., 2005). These novel MTAs associated with SHI have the potential to increase the efficiency of breeding programs aimed at optimizing GY and its major components in wheat upon validation.

We detected 12 MTAs associated with GY distributed on four chromosomes: 2A, 3A, 3B, and 5D with PVEs ranging from 14 to 30% (**Table 2**, **Figure S3.2**). Previous reports have also found MTAs for GY on wheat chromosomes 2A (Qadir et al., 2014; Sukumaran et al., 2017; Wang et al., 2017; Ullah, 2018), 3A (Neumann et al., 2011; Bordes et al., 2014; Hoffstetter et al., 2016; Sehgal et al., 2017), 3B (Sukumaran et al., 2015; Ogbonnaya et al., 2017; Sukumaran et al., 2017), and 5D (Qaseem et al., 2018). Identification of MTAs associated with GY on the same chromosome as mentioned in past studies adds higher confidence to these MTAs. For instance, several studies have reported chromosome 3A as a hotspot for MTAs associated with GY (Ali et al., 2011; Neumann et al., 2011; Bordes et al., 2014; Edae et al., 2014; Hoffstetter et al., 2016; Ogbonnaya et al., 2017; Sehgal et al., 2017; Sukumaran et al., 2017; Wang et al., 2017). Past literature reported pleiotropic markers associated with GY, and yield related traits in spring wheat (Edae et al., 2014; Sukumaran and Yu, 2014). In this study, GY shared common MTAs with HI (S3A_12525847, S3A_625236830), GN (S3A_625236830), TGW (S3A_12554700), and GFR (S3A_625236830, S3B_605293365) which explained 15–29% of trait phenotypic variation. These are potential new targets for multi-trait improvement and marker assisted breeding. Eight out of 12 MTAs for GY were on chromosome 3A, of which six MTAs were within 400 kb of each other with PVEs ranging from 28 to 30%. Three MTAs for GY were found in two genes encoding sulfotransferase and the XH/XS domain protein (**Table 4**). Both genes had a predicted function related to abiotic stress response (Qin et al., 2009; Yokotani et al., 2009).

For GN, we identified 10 significant MTAs distributed on chromosomes 1D, 2B, 3A, 4B, 4D, 5A, 6A, and 6B (**Table 2**, **Figure S3.3**). Earlier studies have identified MTAs associated with GN on chromosomes 2B (Sukumaran et al., 2018), 3A (Grogan, 2015), 4B (Grogan, 2015), 5A (Sukumaran et al., 2015; Lozada et al., 2017; Sukumaran et al., 2018), 6A (Grogan, 2015; Ogbonnaya et al., 2017; Sukumaran et al., 2018), and 6B (Grogan, 2015). The two MTAs on chromosome 1D (S1D_108803053) and 4D (S4D_347074837) are potentially novel MTAs associated with GN. S1D_108803053 had pleiotropic effect for GN and SF (−log10(P) ≤ 3.5) indicating a genetic association between these traits (**Table S4**). Five out of 10 MTAs for GN were located in five genes with different functional annotations. One MTA on chromosome 2B (S2B_692461029) is annotated as an RNA-binding family protein and has been reported to have a role in response to high temperature (Qin et al., 2008). This MTA also had pleiotropic effect for GN and SF (−log10(P) ≤ 3.5) thus making it a stronger candidate for future functional validation studies (**Table S4**). One MTA on chromosome 6B (S6B_564061696) within gene TraesCS6B01G316000 is annotated as a cellulose synthase gene and had a predicted function related to abiotic stress response (Zhu et al., 2010) (**Table 4**). One MTA (S5A_605144047) on chromosome 5A has a pleiotropic effect for GN, SF, and TGW and was found in gene (TraesCS5A01G416300) which is annotated as inosine-5'-monophosphate dehydrogenase. This gene had been previously reported as one of the differentially expressed genes in response to drought stress in pearl millet (Choudhary and Padaria, 2015).

We reported 12 MTAs associated with HI on chromosomes 3A, 5A, and 5B (**Table 2**, **Figure S3.8**). Past studies have also reported MTAs associated with HI on chromosomes 3A (Awad, 2015; Bhatta et al., 2018), 5A (Awad, 2015; Qaseem et al., 2018), and 5B (Bhatta et al., 2018). Three MTAs (S5B_421762510, S5B_421762511 and S5B_421762531) within 20 bp of each other were detected on chromosome 5B across multiple environments with a functional annotation of carboxypeptidase (**Table 4**). Carboxypeptidase is a protease enzyme with diverse functions ranging from catabolism to stress response. Serine carboxypeptidase, a class of carboxypeptidase has been reported to be a heat stress responsive protein in wheat (Kamal et al., 2010) and rice (Majoul et al., 2003).

Twenty-eight MTAs were associated with TGW on chromosomes 1B, 2B, 3A, 4A, 5A, 6B, 7A, and 7D with PVEs ranging from 24 to 31% (**Table 2**, **Figure S3.4**). MTAs for TGW on these same chromosomes have been reported by other researchers: 1B (Sukumaran et al., 2017; Ma et al., 2018), 2B (Neumann et al., 2011; Lozada et al., 2017; Sukumaran et al., 2017; Bhatta et al., 2018; Liu et al., 2018; Ma et al., 2018; Sukumaran et al., 2018; Ullah, 2018), 3A (Zanke et al., 2014; Lozada et al., 2017; Sun et al., 2017; Bhatta et al., 2018; Ma et al., 2018), 4A (Bhatta et al., 2018; Liu et al., 2018; Ma et al., 2018), 5A (Sukumaran et al., 2015; Sukumaran et al., 2017; Wang et al., 2017; Ma et al., 2018; Qaseem et al., 2018), 6B (Sukumaran et al., 2017; Sun et al., 2017), 7A (Sukumaran et al., 2017; Sun et al., 2017; Wang et al., 2017; Liu et al., 2018; Ma et al., 2018; Qaseem et al., 2018), and 7D (Sukumaran et al., 2017; Bhatta et al., 2018; Liu et al., 2018; Qaseem et al., 2018; Ullah, 2018). Nineteen out of 28 MTAs were identified on chromosomes 1B, 2B, 4A, 5A, 6B, 7A, and 7D within 11 genes (**Table 4**) with different functions. Three MTAs on chromosome 2B (S2B_93702429, S2B_93702446, S2B_93702460) within 31 bp of each other were present within gene TraesCS6B01G199000. This gene is annotated as heat shock protein. Heat shock proteins are well known for plant response to thermotolerance and are usually undetectable at optimal growing temperatures (Wang et al., 2004). We found several other MTAs whose annotations suggest different roles including biotic and abiotic stress response (CASP-like protein, really interesting new gene finger and CHY zinc finger protein, histone H2B, transmembrane protein, wall-associated receptor kinase), disease resistance (flavonoid 3'-hydroxylase), and pollen sterility (adenine nucleotide alpha hydrolases-like superfamily protein) (Mok and Mok, 2001; Lenka et al., 2011; Xin et al., 2012; Zhu et al., 2012; Yang et al., 2015).

For GFR, we identified five MTAs on chromosomes 2A, 3A, 3B, and 5D with phenotypic variation explained (PVEs) ranging from 19 to 25% (**Table 2**, **Figure S3.5**).

The 11 MTAs responsible for SPK were detected on chromosomes 2A, 3D, 5A, 6A, 6B, and 7A (**Table 2**, **Table S2** and **Figure S3.6**). MTAs for SPK have been detected on chromosomes 2A (Grogan, 2015; Qaseem et al., 2018), 3D (Qaseem et al., 2018), 5A (Ogbonnaya et al., 2017; Qaseem et al., 2018), 6A (Qaseem et al., 2018), and 7A (Ogbonnaya et al., 2017). In this study, we discovered four new MTAs for SPK on chromosome 6B. These MTAs were linked to S6B_148823518, S6B_484538781, S6B_699027892, and S6B_699027892 which are found in genes annotated as ATP-dependent DNA helicase MER3-like protein, lysophospholipid acyltransferase, and receptor-like kinase respectively (**Table 4**). Receptor-like kinase proteins have been reported to have a role in heat stress response (Shen et al., 2015). MTAs detected on chromosomes 2A, have gene annotations for F-box family proteins (TraesCS2A01G479200) suggesting their involvement in biotic and abiotic stress response (Li et al., 2018).

## Conclusion

This study identified a large number of MTAs in soft winter wheat for SF, SHI, GY, and other yield-related traits under hot environments. Stable loci across environments and pleiotropic markers controlling multiple traits were identified. We also identified candidate genes affecting several important biological processes in plants including response to heat stress. Nonetheless, a limitation of this study was that only 236 lines were evaluated in two locations. Further validation of these MTAs in larger populations and more environments can have strong implications for gene discovery and marker-assisted breeding under high temperature stress environments to improve sink strength. Identifying regulatory loci associated with traits like SF and SHI can help us understand the complex nature of assimilate partitioning under current and future climate conditions. These findings will assist to utilize the identified genetic loci associated with SF and SHI to develop the best ideotypes that maximize conversion of enhanced carbon capture and biomass growth for GY through exploration of new genetic variation in related traits and genetic markers associated with these traits.

## Data Availability Statement

The SNP data generated for this study can be found in the NCBI using accession number PRJNA578088 (https://www.ncbi.nlm.nih.gov//bioproject/PRJNA578088).

## Author Contributions

MAB conceived and designed the study. SP completed the study and data analysis and wrote the manuscript. JG, KR, and MB contributed to statistical analysis and editing of the manuscript. GB, PA, and AB performed marker analysis and SNP calling. RM, EM, MM, SA, SG, B-KB, and ARB edited the manuscript. JK, MH, DS, MA, and JG collected the phenotypic data on association panel and edited the manuscript.

## Funding

This research was funded by UF/IFAS early career award.

## Conflict of Interest

The authors declare that the research was conducted in the absence of any commercial or financial relationships that could be construed as a potential conflict of interest.
