# Supplementary material for: Understanding the Genetic Basis of Spike Fertility to Improve Grain Number, Harvest Index, and Grain Yield in Wheat Under High Temperature Stress Environments
Source: Front Plant Sci. 2019 Nov 29;10:1481. doi: 10.3389/fpls.2019.01481 (PMC6895025; doi:10.3389/fpls.2019.01481)
Supplement: Supplementary file 1 [file Table_1.docx]

Supplementary Material

# Supplementary Figures

#
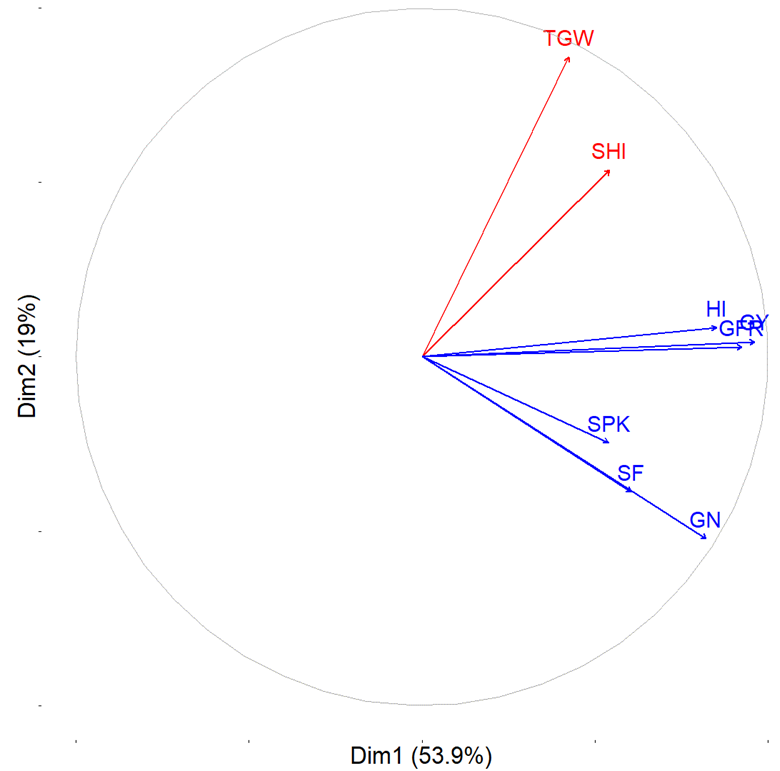


**A**

#
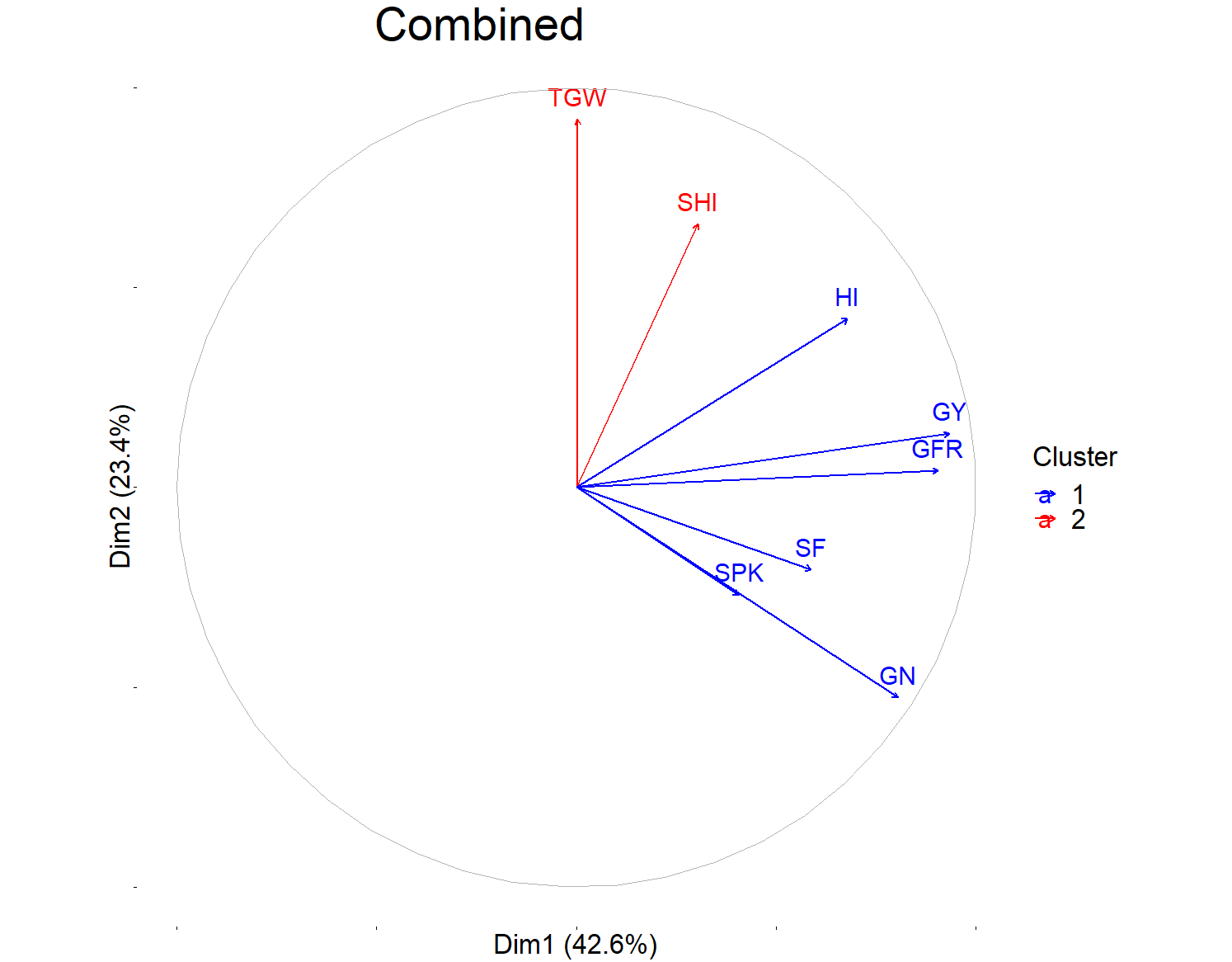


**B**

**C**

#
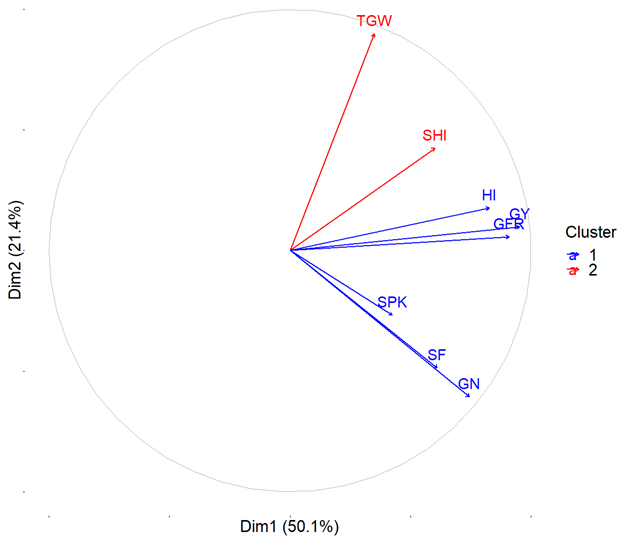


# Figure S1: Principal component bi-plot analysis of measured traits for SWAMP using best linear unbiased estimates in three datasets: Citra (A-BLUEC), Quincy (B-BLUEQ), combined (C-BLUEA). SF, spike fertility (grains g^-1^ chaff weight); GY, grain yield (kg h^-1^); GN, grain number m^-²^; TGW, thousand grain weight (g); GFR, grain filling rate (kg h^-1^ days); SPK, number of spikes m^-²^; SHI, spike harvest index; HI, harvest index.

#
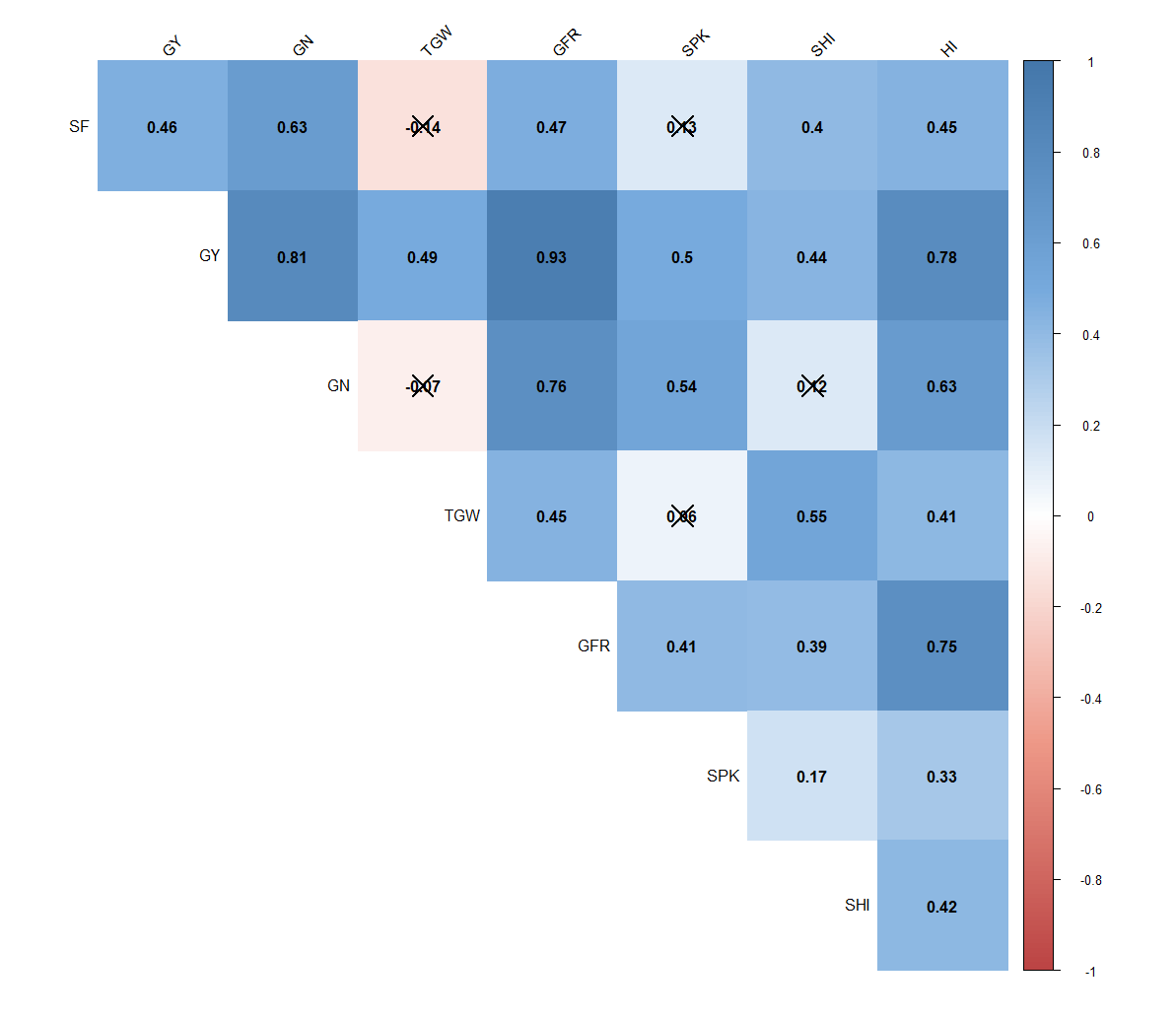


**A**

**B**

#
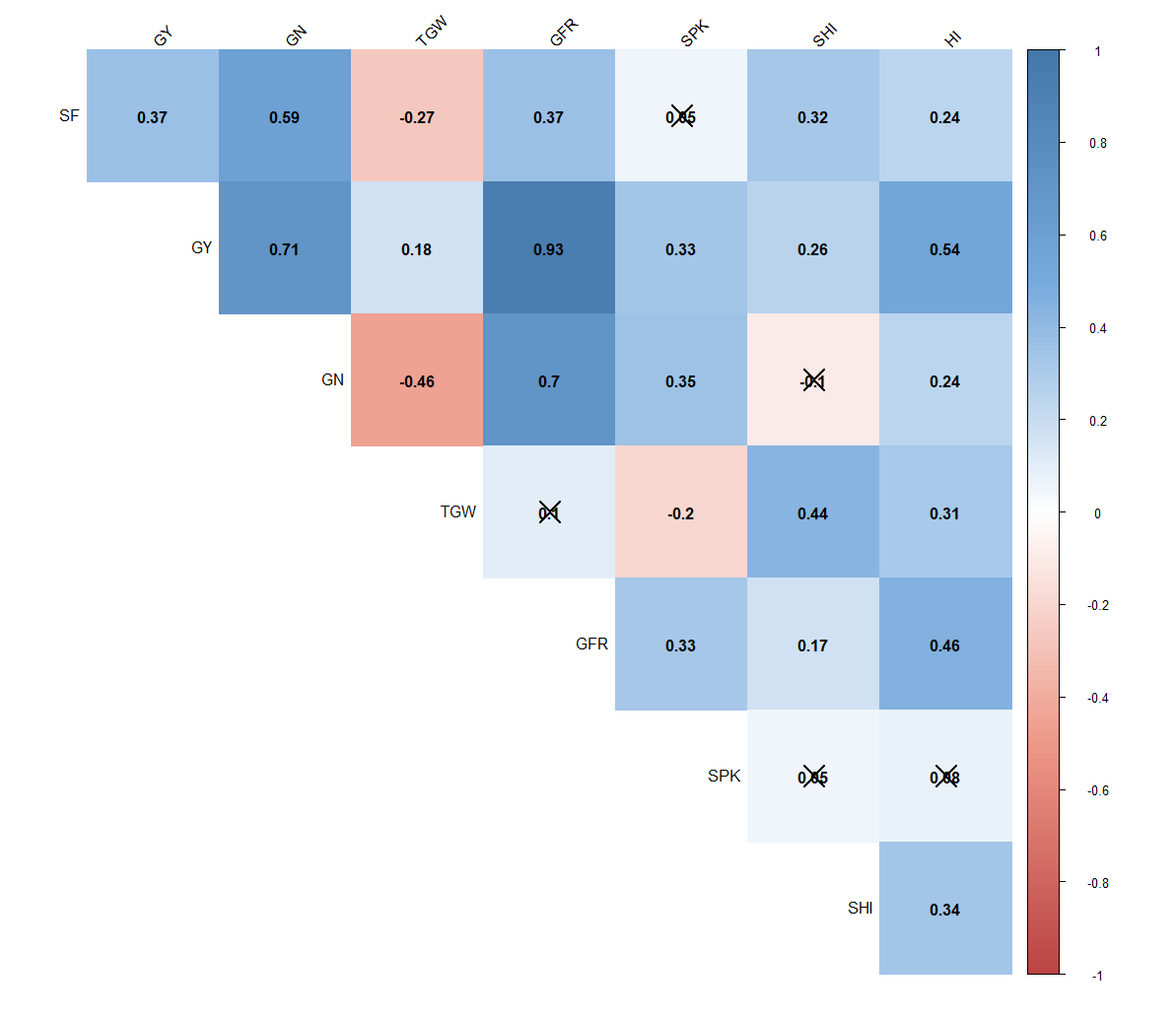


#
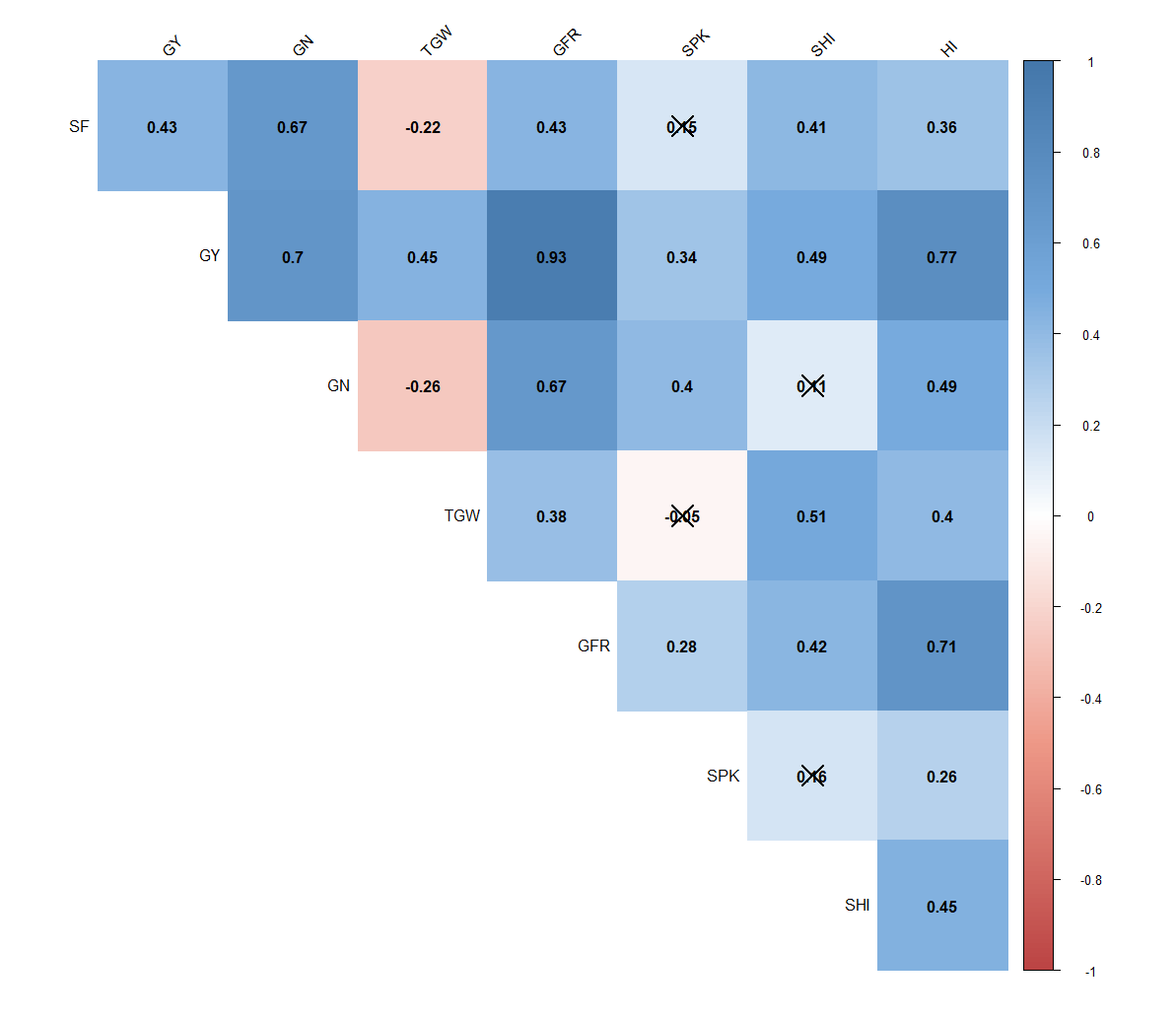


**C**

# Figure S2: Pearson’s correlation coefficient between phenotypic traits for SWAMP using best linear unbiased estimates in three datasets: Citra (A-BLUEC), Quincy (B-BLUEQ), combined (C-BLUEA). Cross mark indicates no significant correlation at p<0.01 level. SF, spike fertility (grains g^-1^ chaff weight); GY, grain yield (kg h^-1^); GN, grain number m^-²^; TGW, thousand grain weight (g); GFR, grain filling rate (kg h^-1^ days); SPK, number of spikes m^-²^; SHI, spike harvest index; HI, harvest index.

#
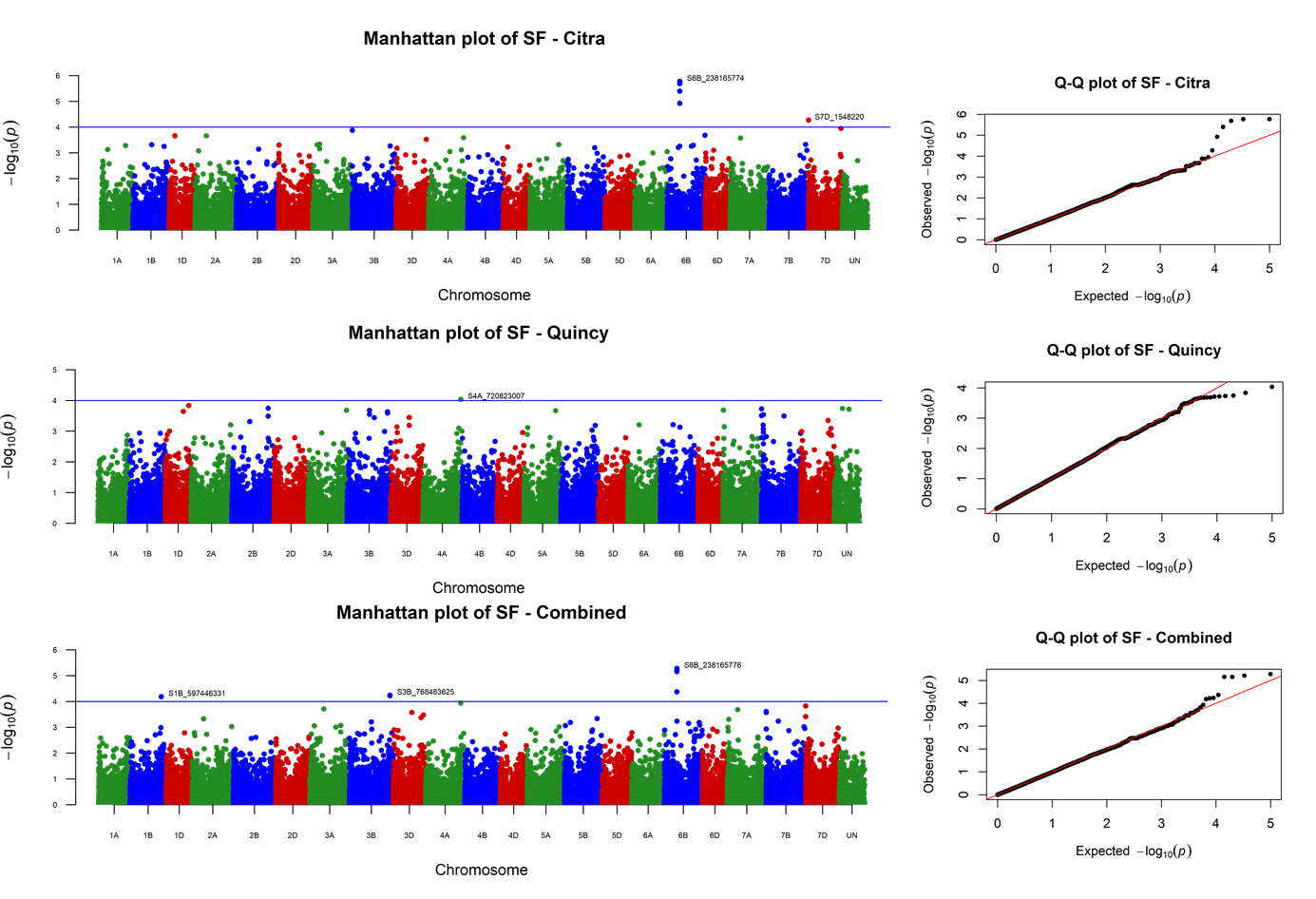


# Figure S3.1. Manhattan plot (left) and quantile-quantile plots (right) showing genome-wide SNP loci associated with spike fertility (SF) in SWAMP using three datasets based on best linear unbiased estimates values: Citra (BLUEC, upper), Quincy (BLUEQ, middle) and combined (BLUEA, lower). The horizontal blue line in manhattan plot represents the expected value with a uniform suggestive genome wide significance threshold [-log10(P) ≤ 4.00].

#
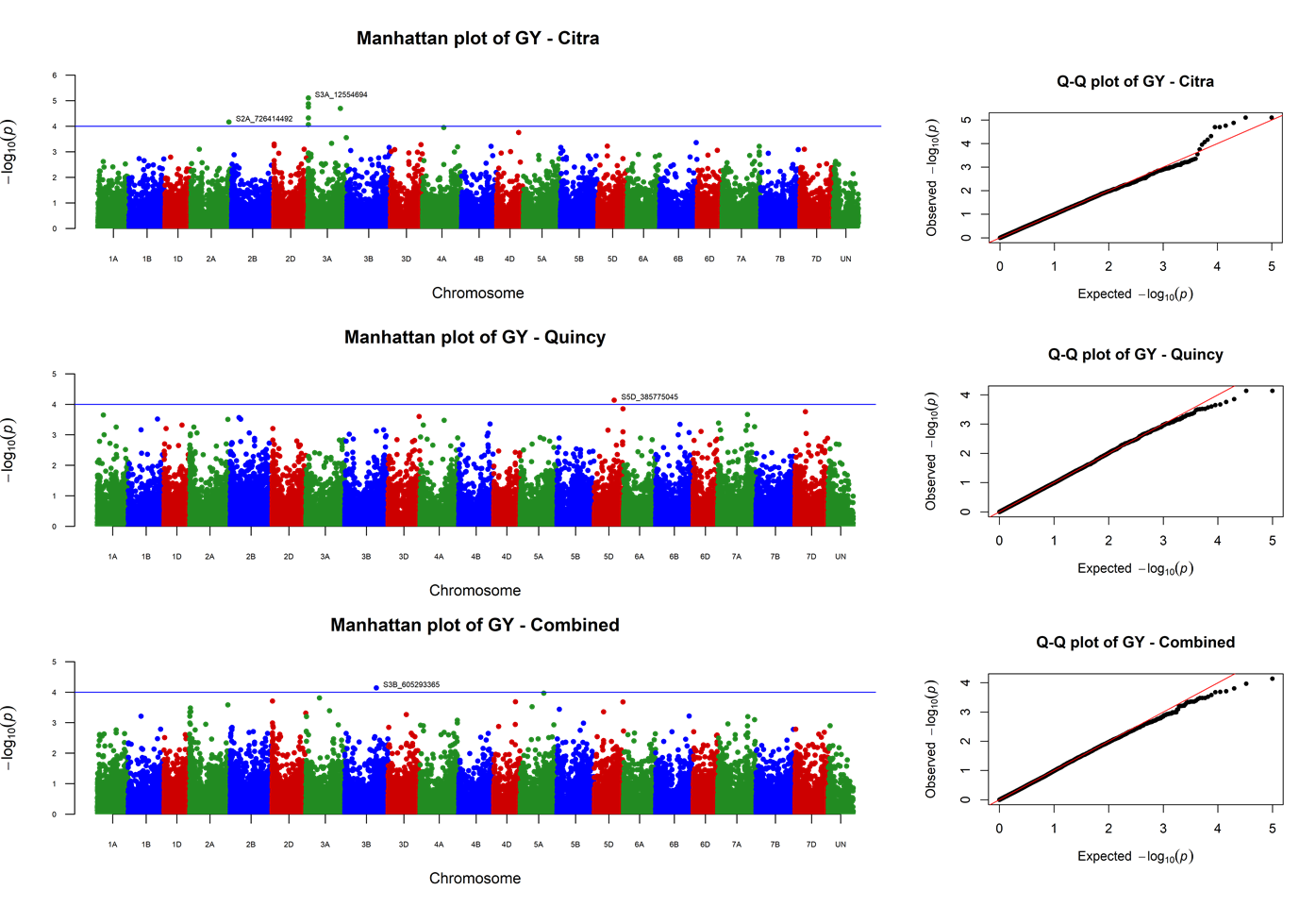


# Figure S3.2. Manhattan plot (left) and quantile-quantile plots (right) showing genome-wide SNP loci associated with grain yield (GY) in SWAMP using three datasets based on best linear unbiased estimates values: Citra (BLUEC, upper), Quincy (BLUEQ, middle) and combined (BLUEA, lower). The horizontal blue line in manhattan plot represents the expected value with a uniform suggestive genome wide significance threshold [-log10(P) ≤ 4.00].

#
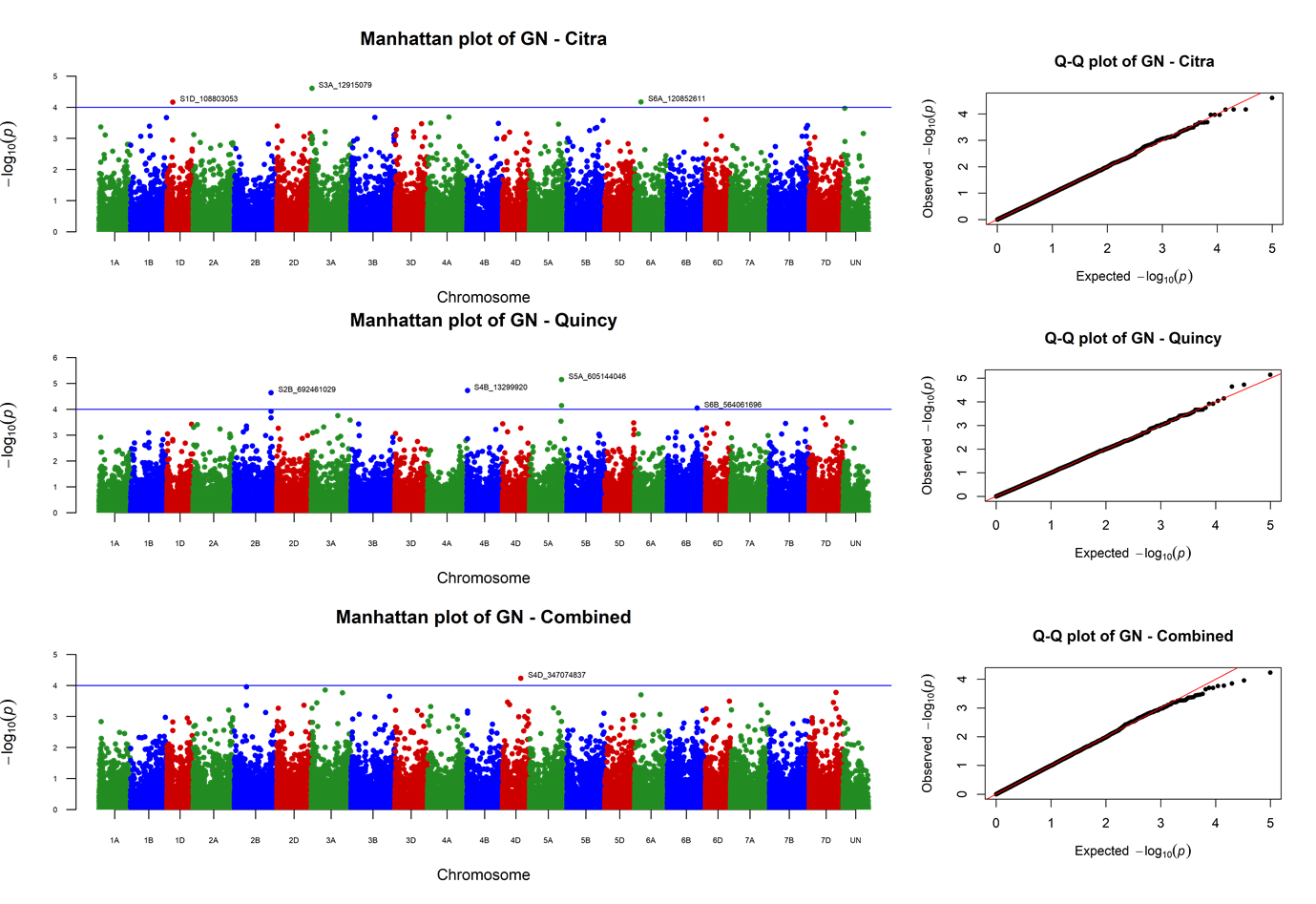


**Figure S3.3.** Manhattan plot (left) and quantile-quantile plots (right) showing genome-wide SNP loci associated with grain number (GN) in SWAMP using three datasets based on best linear unbiased estimates values: Citra (BLUEC, upper), Quincy (BLUEQ, middle) and combined (BLUEA, lower). The horizontal blue line in manhattan plot represents the expected value with a uniform suggestive genome wide significance threshold [-log10(P) ≤ 4.00].

#
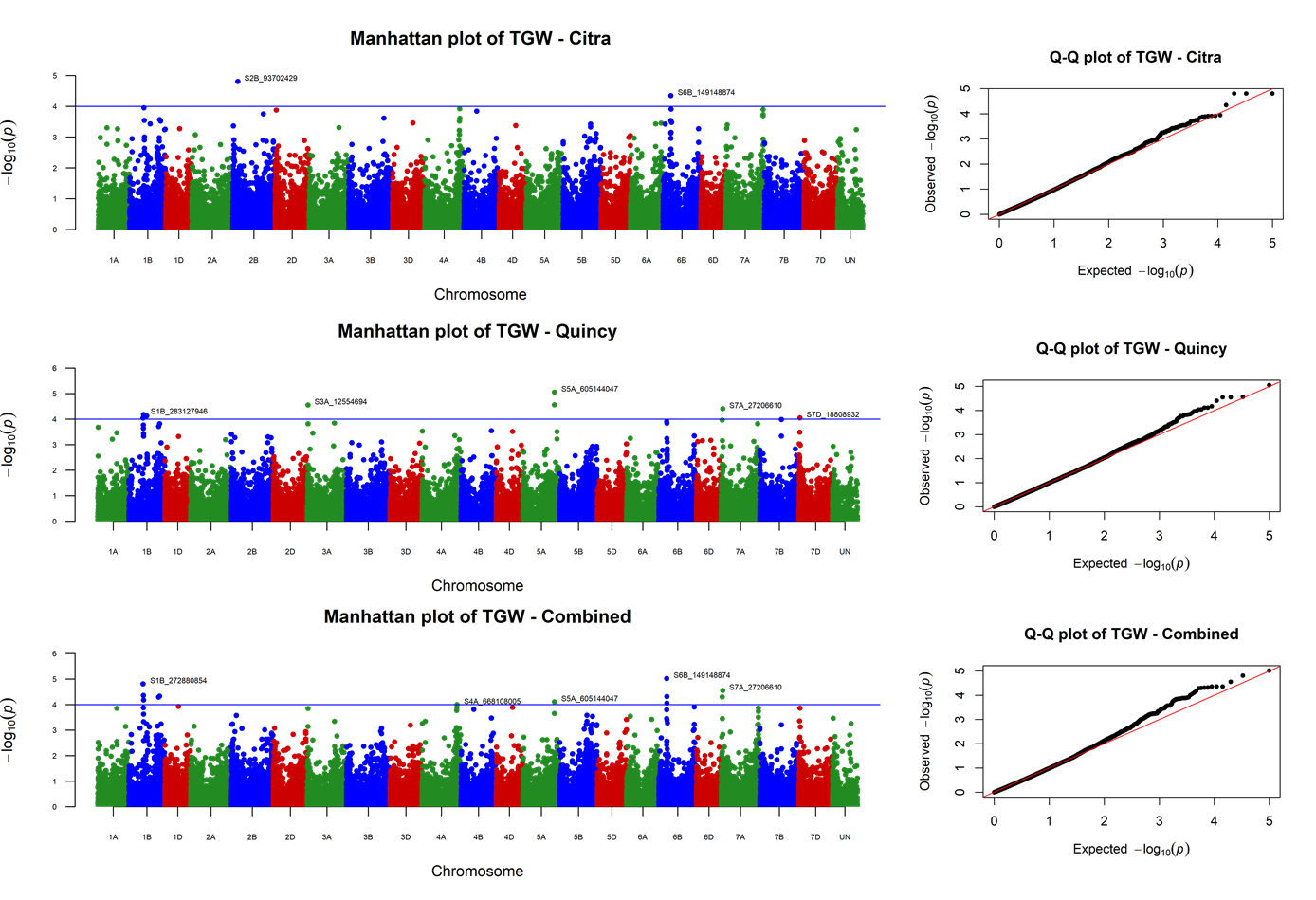


# Figure S3.4. Manhattan plot (left) and quantile-quantile plots (right) showing genome-wide SNP loci associated with thousand grain weight (TGW) in SWAMP using three datasets based on best linear unbiased estimates values: Citra (BLUEC, upper), Quincy (BLUEQ, middle) and combined (BLUEA, lower). The horizontal blue line in manhattan plot represents the expected value with a uniform suggestive genome wide significance threshold [-log10(P) ≤ 4.00].

#
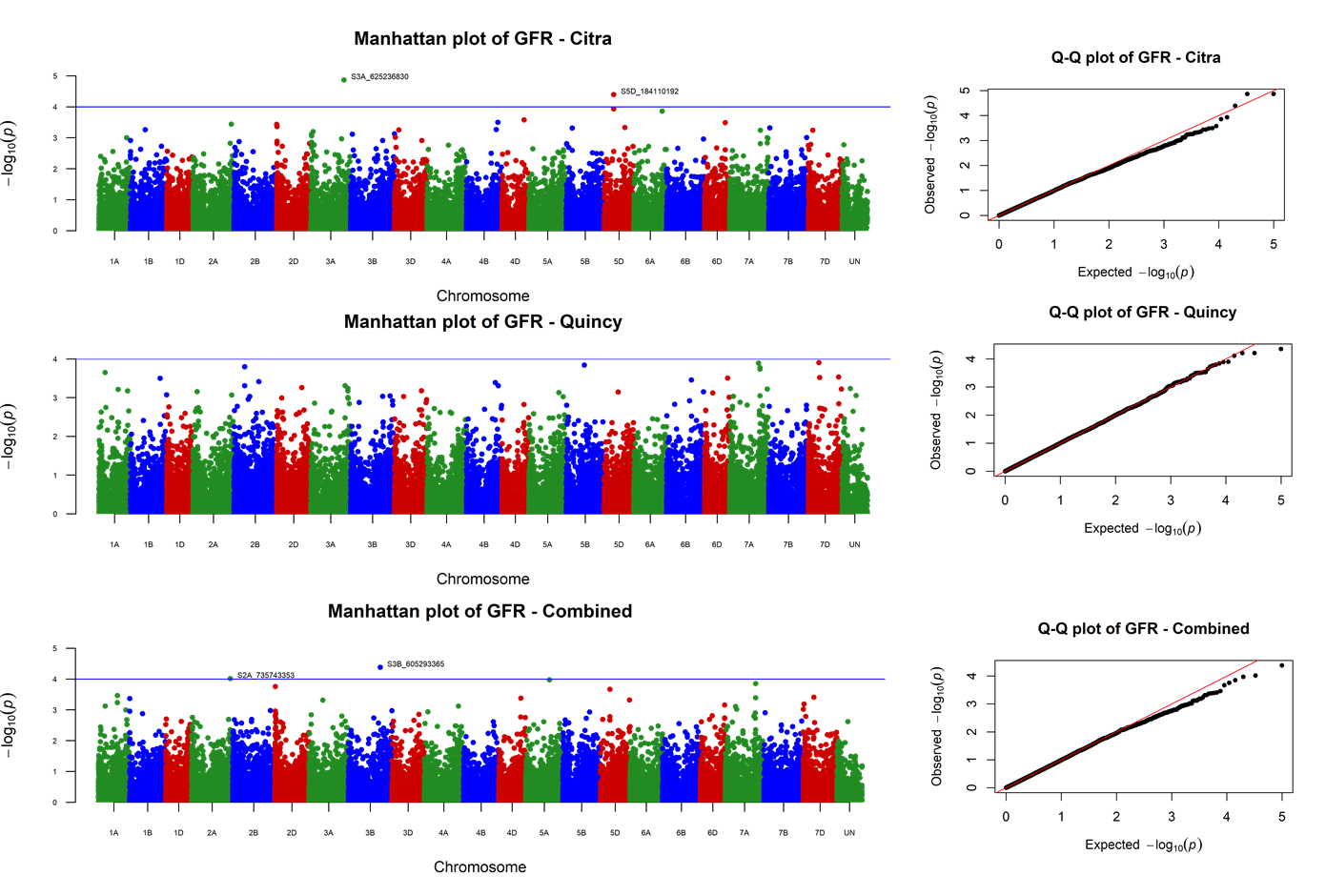


# Figure S3.5. Manhattan plot (left) and quantile-quantile plots (right) showing genome-wide SNP loci associated with grain filling rate (GFR) in SWAMP using three datasets based on best linear unbiased estimates values: Citra (BLUEC, upper), Quincy (BLUEQ, middle) and combined (BLUEA, lower). The horizontal blue line in manhattan plot represents the expected value with a uniform suggestive genome wide significance threshold [-log10(P) ≤ 4.00].

#
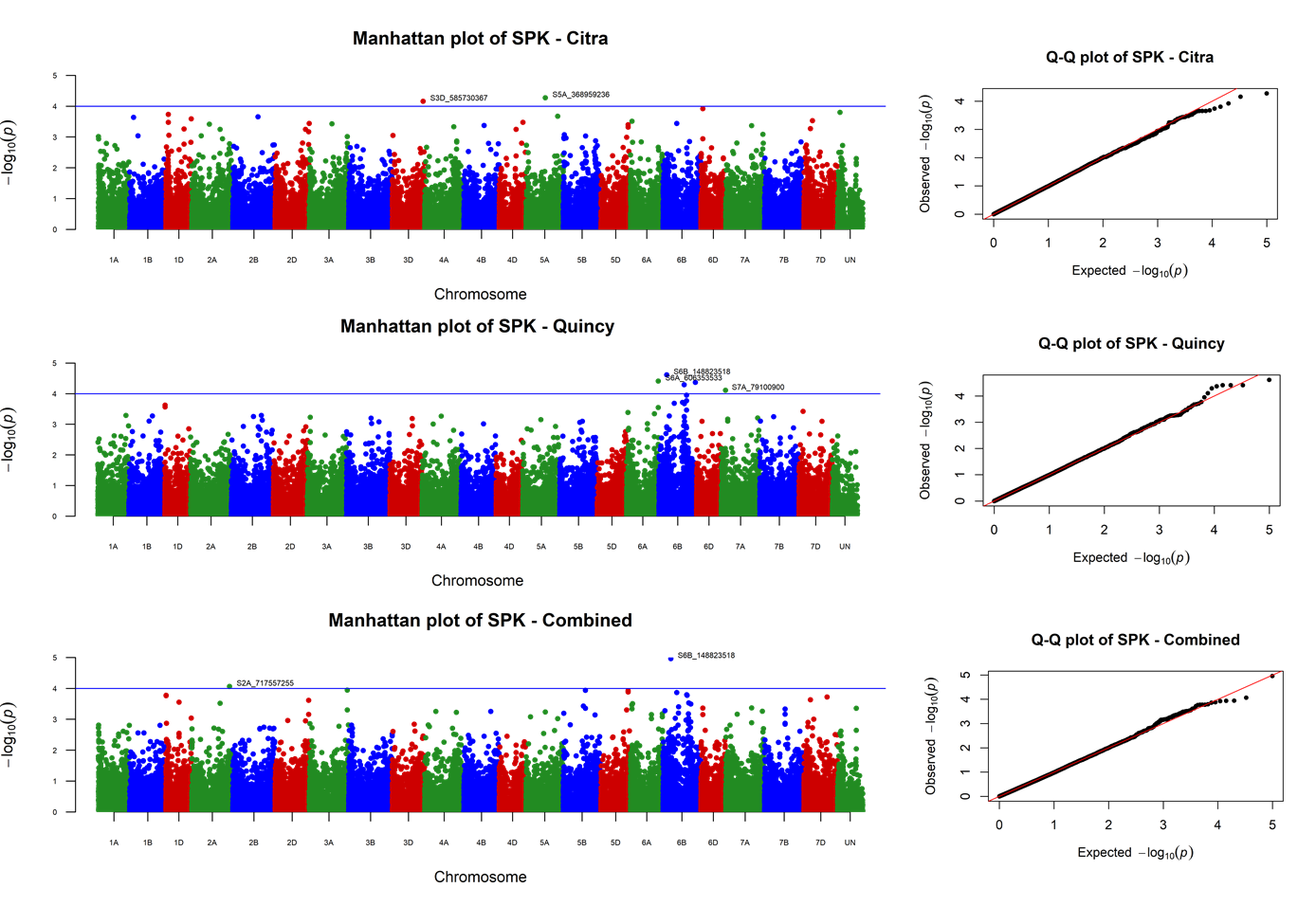


# Figure S3.6. Manhattan plot (left) and quantile-quantile plots (right) showing genome-wide SNP loci associated with number of spikes per m2 (SPK) in SWAMP using three datasets based on best linear unbiased estimates values: Citra (BLUEC, upper), Quincy (BLUEQ, middle) and combined (BLUEA, lower). The horizontal blue line in manhattan plot represents the expected value with a uniform suggestive genome wide significance threshold [-log10(P) ≤ 4.00].

#
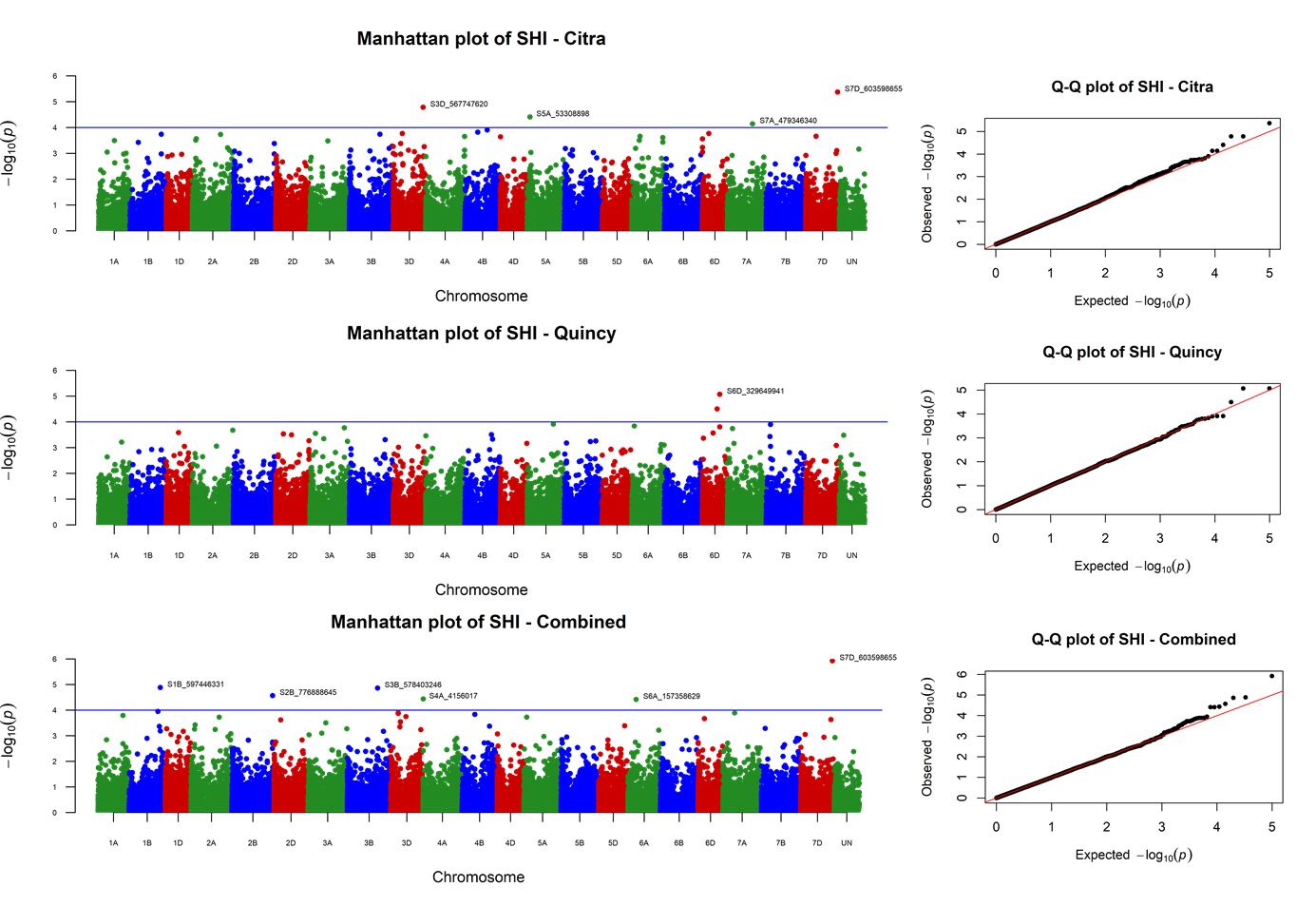


# Figure S3.7. Manhattan plot (left) and quantile-quantile plots (right) showing genome-wide SNP loci associated with spike harvest index (SHI) in SWAMP using three datasets based on best linear unbiased estimates values: Citra (BLUEC, upper), Quincy (BLUEQ, middle) and combined (BLUEA, lower). The horizontal blue line in manhattan plot represents the expected value with a uniform suggestive genome wide significance threshold [-log10(P) ≤ 4.00].

#
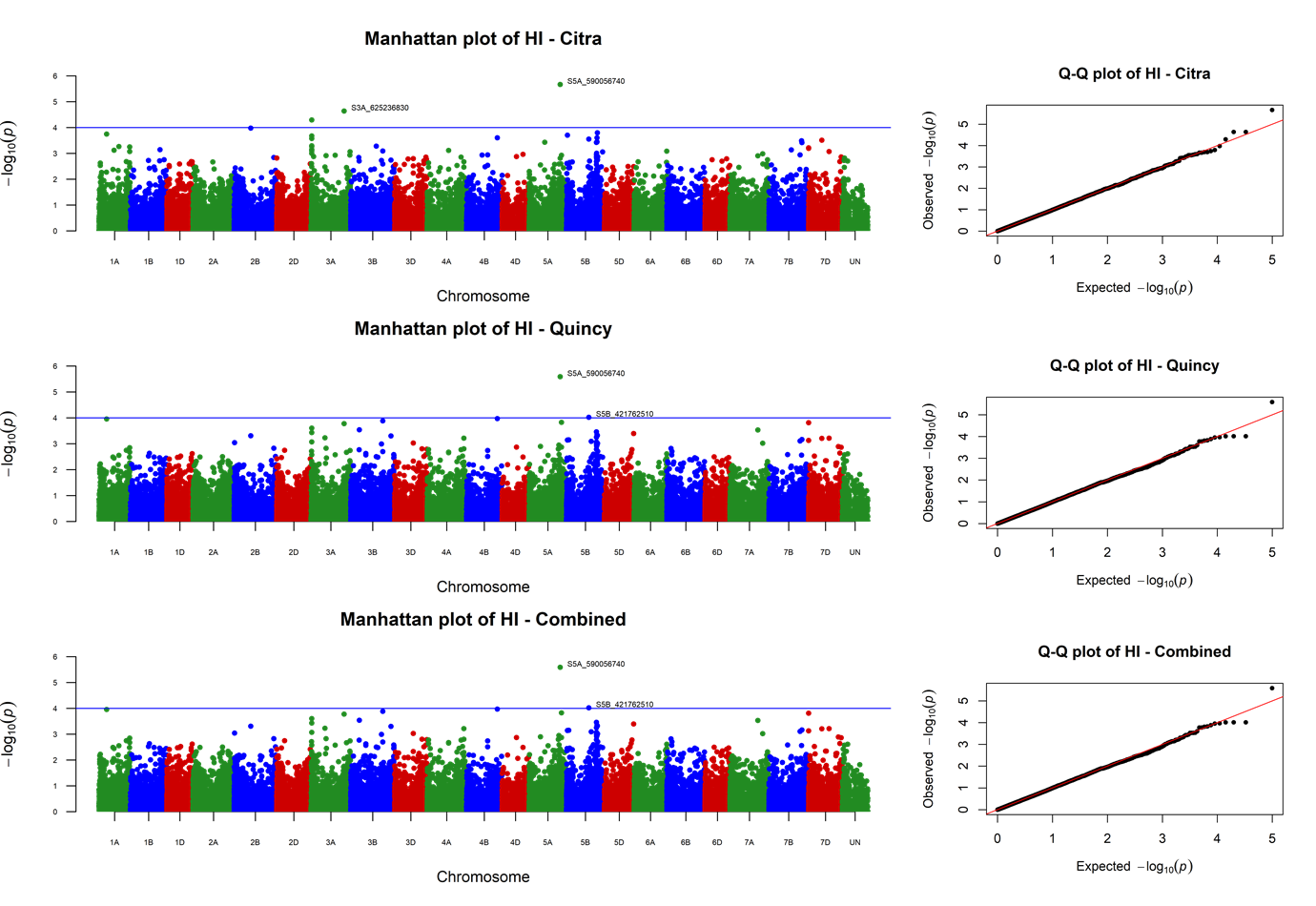


# Figure S3.8. Manhattan plot (left) and quantile-quantile plots (right) showing genome-wide SNP loci associated with HI harvest index in SWAMP using three datasets based on best linear unbiased estimates values: Citra (BLUEC, upper), Quincy (BLUEQ, middle) and combined (BLUEA, lower). The horizontal blue line in manhattan plot represents the expected value with a uniform suggestive genome wide significance threshold [-log10(P) ≤ 4.00].

#
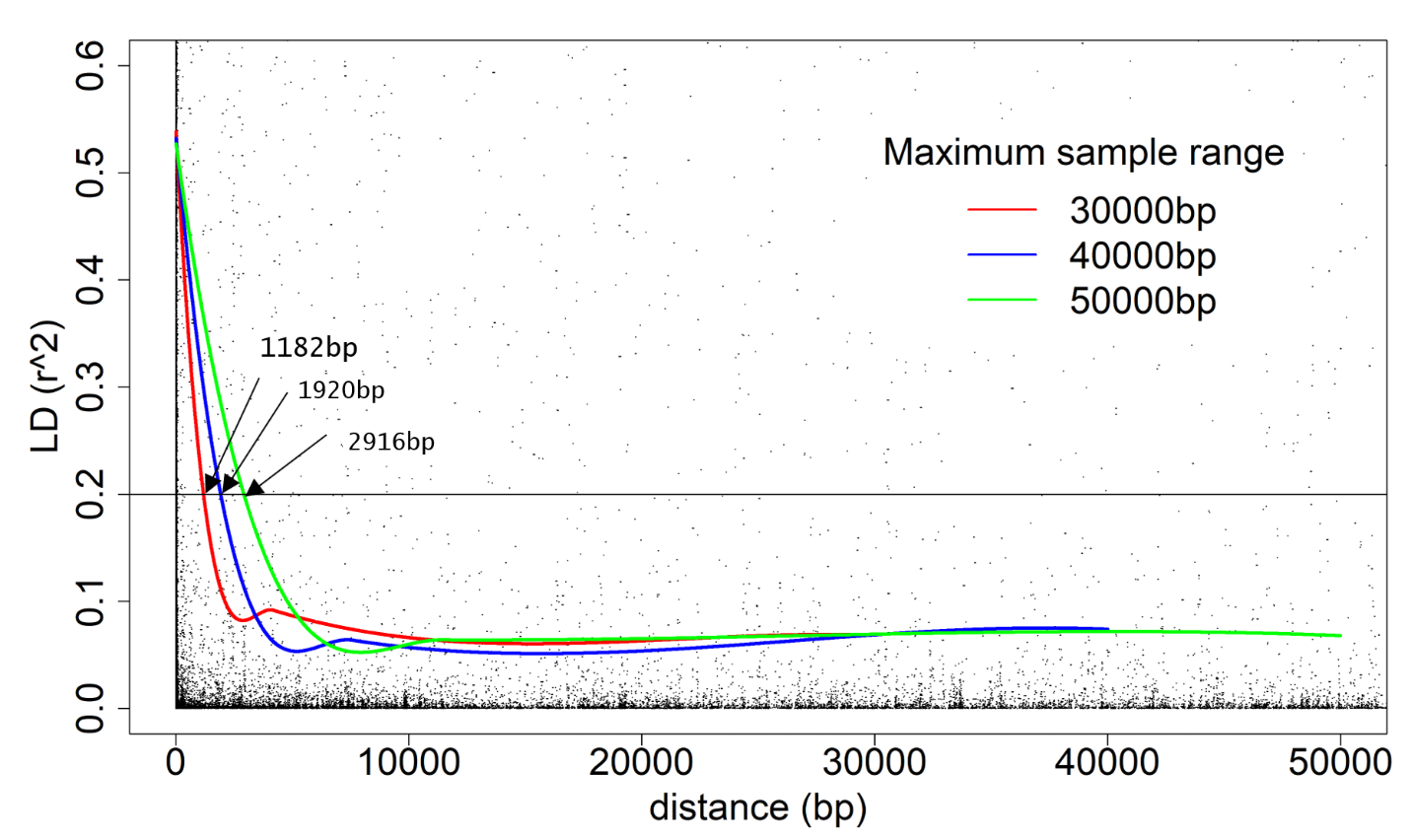


# Figure S4: Linkage disequilibrium represented by the r^2^ against physical distance (in bp) showing LD decay with increasing distances among pairs of marker loci for the whole genomes. LOESS regressions of mean r^2^ between pairs of SNPs vs. physical distance were sampled at 30,000 (red), 40,000 (blue), and 50,000 (green) bp. Grey line represents the critical value beyond which LD is likely caused by physical linkage.


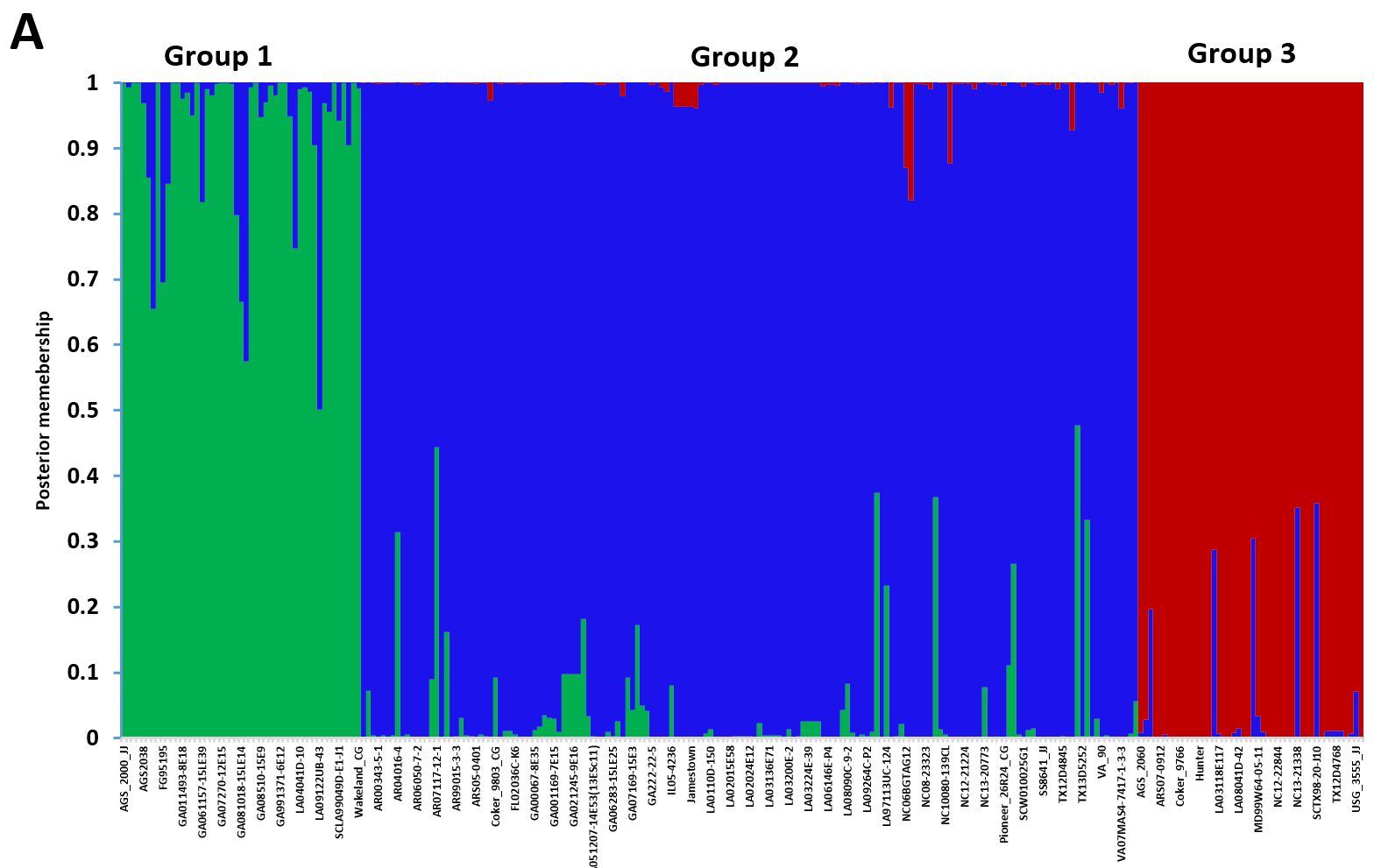


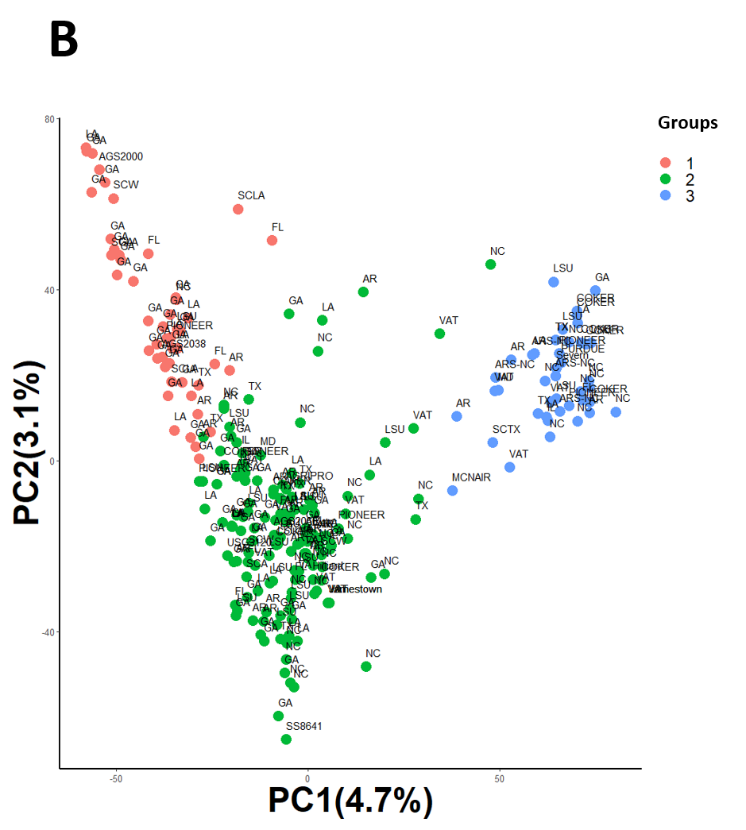


# Figure S5: Population structure of SWAMP based on 27,466 SNPs (A) bar charts showing posterior probabilities of assignment to three groups based on algorithms of discriminant analysis of principal components (DAPC) (B) Population structure among groups inferred from PC analysis. The populations were colored based on the posterior of probability assigned to three genetic groups inferred from DAPC.


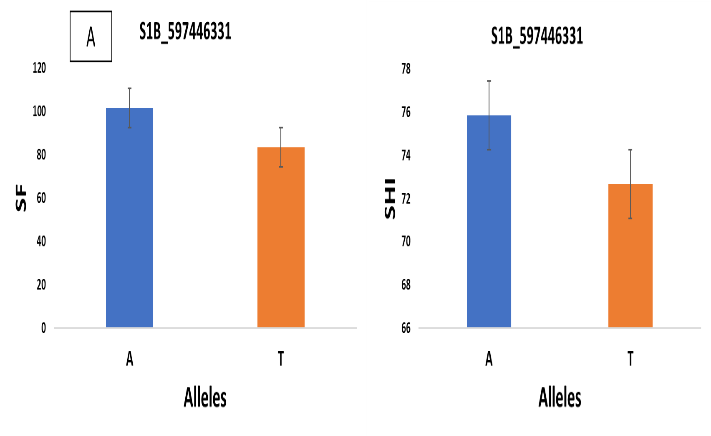

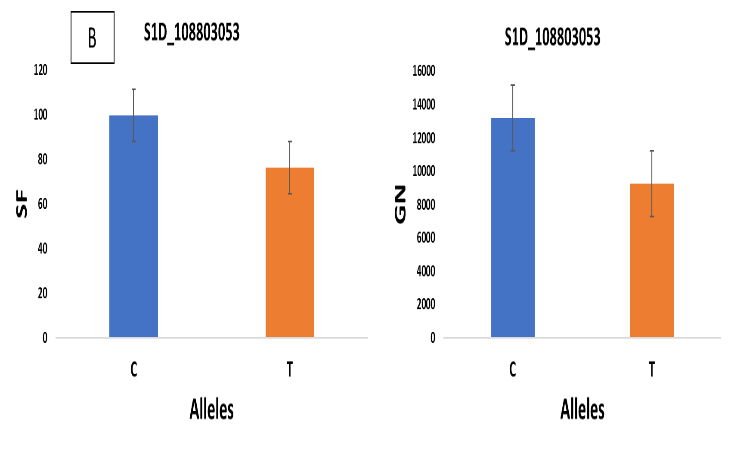


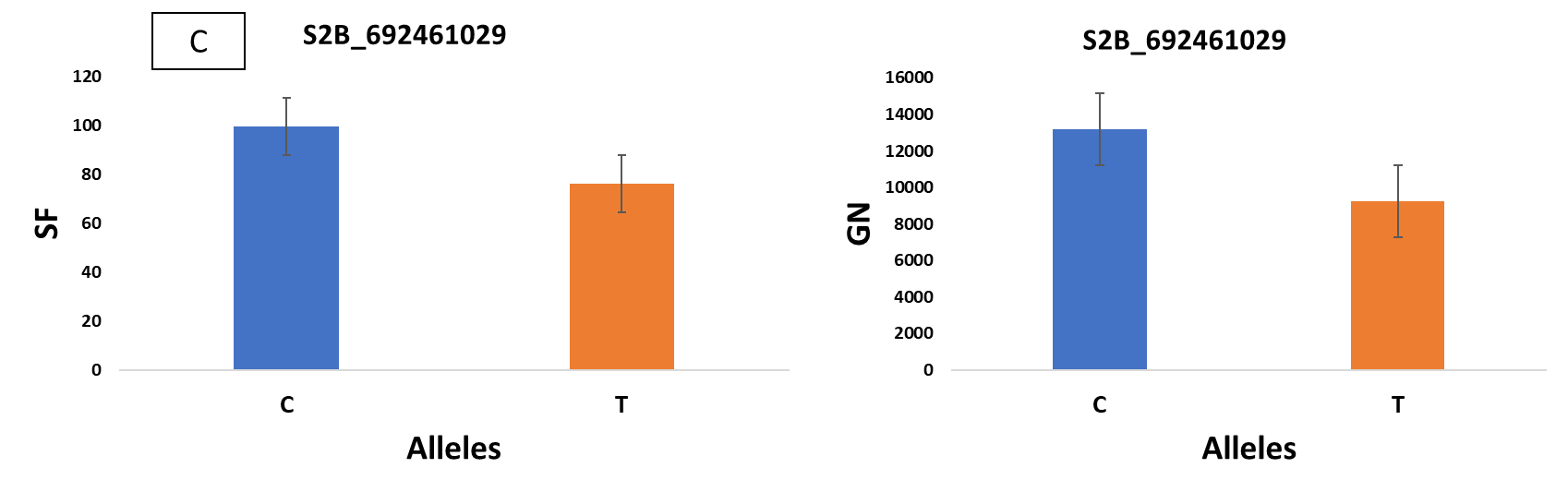

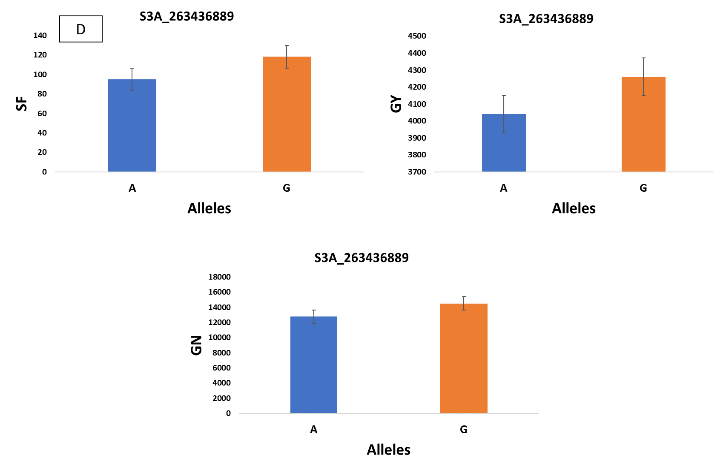


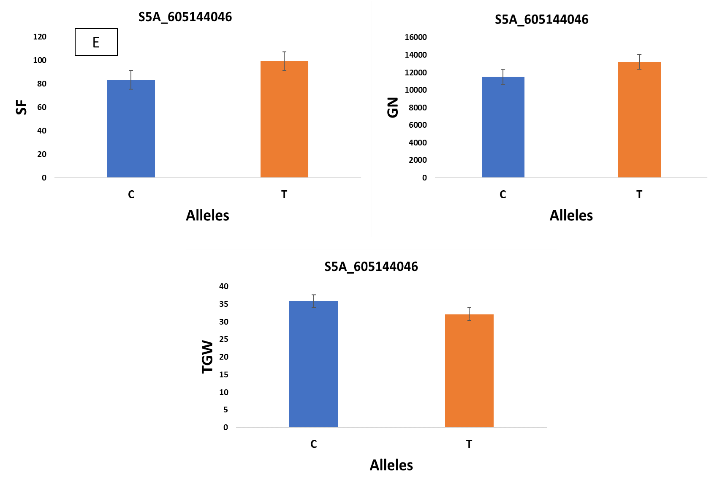

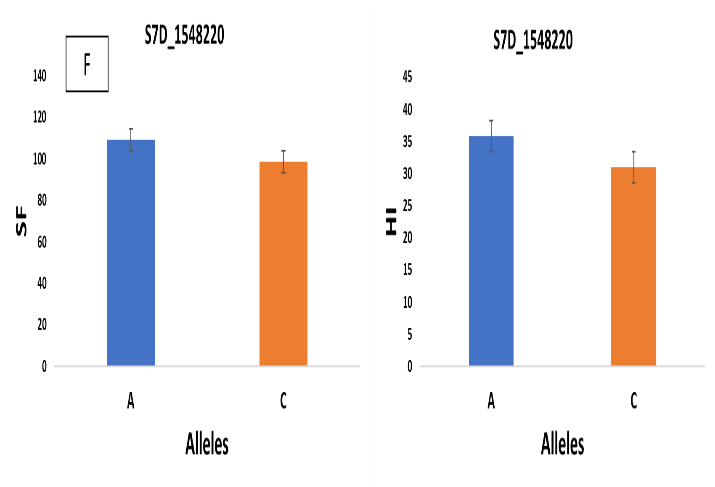


# Figure S6: Different alleles significantly associated with SF and their individual effect on SF, SHI, GY, GN and TGW.
